# From Basic Science to Clinical Practice: A Review of Current Periodontal/Mucogingival Regenerative Biomaterials

**DOI:** 10.1002/advs.202308848

**Published:** 2024-02-21

**Authors:** Angela De Lauretis, Øystein Øvrebø, Mario Romandini, Ståle Petter Lyngstadaas, Filippo Rossi, Håvard Jostein Haugen

**Affiliations:** ^1^ Department of Biomaterials, Institute of Clinical Dentistry, Faculty of Dentistry University of Oslo Oslo 0455 Norway; ^2^ Department of Chemistry, Materials and Chemical Engineering “Giulio Natta” Politecnico di Milano Milan 20133 Italy; ^3^ Department of Periodontology, Institute of Clinical Dentistry, Faculty of Dentistry University of Oslo Oslo 0455 Norway

**Keywords:** bone grafts, hydrogels, periodontal regeneration, platelet concentrates, putties

## Abstract

Periodontitis is a dysbiosis‐driven inflammatory disease affecting the tooth‐supporting tissues, characterized by their progressive resorption, which can ultimately lead to tooth loss. A step‐wise therapeutic approach is employed for periodontitis. After an initial behavioral and non‐surgical phase, intra‐bony or furcation defects may be amenable to regenerative procedures. This review discusses the regenerative technologies employed for periodontal regeneration, highlighting the current limitations and future research areas. The search, performed on the MEDLINE database, has identified the available biomaterials, including biologicals (autologous platelet concentrates, hydrogels), bone grafts (pure or putty), and membranes. Biologicals and bone grafts have been critically analyzed in terms of composition, mechanism of action, and clinical applications. Although a certain degree of periodontal regeneration is predictable in intra‐bony and class II furcation defects, complete defect closure is hardly achieved. Moreover, treating class III furcation defects remains challenging. The key properties required for functional regeneration are discussed, and none of the commercially available biomaterials possess all the ideal characteristics. Therefore, research is needed to promote the advancement of more effective and targeted regenerative therapies for periodontitis. Lastly, improving the design and reporting of clinical studies is suggested by strictly adhering to the Consolidated Standards of Reporting Trials (CONSORT) 2010 statement.

## Graphical abstract

This review comprehensively examines regenerative technologies in periodontal therapy, exploring biomaterials' composition, action mechanisms, and clinical applications. It critically assesses the quality of related clinical trials and discusses future research directions. The paper highlights the necessity for functional regeneration and the potential of emerging bio‐inspired materials, emphasizing the evolving landscape of periodontal treatment methodologies.

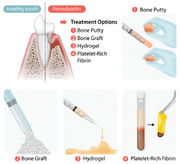

## 1 Introduction

Periodontitis is a chronic multifactorial inflammatory disease associated with dysbiotic dental biofilms and characterized by progressive destruction of the tooth‐supporting apparatus.^[^ ^1^ ^]^ It occurs when untreated gingivitis progresses to the loss of the gingiva, bone, and periodontal ligament, which creates the deep periodontal “pockets” that are a hallmark of the disease.^[^ ^2^ ^]^

If untreated, periodontitis may result in extensive tooth loss, leading to masticatory dysfunction and nutritional compromise, aesthetic impairment, altered speech, low self‐esteem, and a poorer overall quality of life.^[^ ^3^, ^4^, ^5^ ^]^ In 2016, severe periodontitis ranked as the 11th most prevalent condition worldwide according to the Global Burden of Disease Study.^[^ ^6^ ^]^ The prevalence of periodontal disease ranges from 20% to 50% of the global population, with a notable increase starting in the 30–40 age group.^[^ ^7^, ^8^, ^9^ ^]^ Furthermore, periodontitis has a significant global impact, resulting in a cost of 54 billion USD of lost productivity and 3.5 million years lived with disability.^[^ ^6^, ^10^ ^]^ Risk factors for periodontitis include smoking, diabetes, socio‐economic determinants, incorrect lifestyles, and genetic factors.^[^ ^11^, ^12^, ^13^, ^14^, ^15^, ^16^, ^17^, ^18^, ^19^, ^20^, ^21^ ^]^ Apart from its oral sequelae, periodontitis has been regarded as a possible risk factor for several systemic diseases, including cardiovascular diseases, diabetes, Alzheimer's disease, respiratory diseases, cancer, and even mortality.^[^ ^7^, ^22^, ^23^, ^24^, ^25^, ^26^, ^27^, ^28^, ^29^, ^30^ ^]^

**Figure 1:**
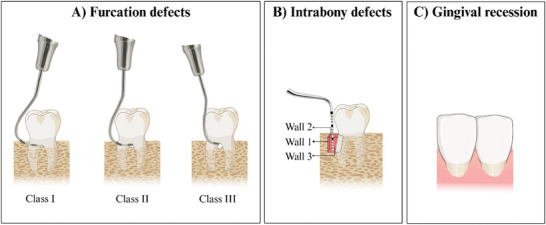
Representation of periodontal defects. A) Furcation defects: Class I (< 3 mm); Class II (≥ 3 mm, without encompassing the total furcation width); Class III (total furcation width). B) Intrabony defects: 1‐wall, 2‐wall, and 3‐wall according to the number of residual bony walls. C) Gingival recession with root exposure.

A step‐wise therapeutic approach is employed for the treatment of periodontitis.^[^ ^31^ ^]^ After a behavioral and a non‐surgical phase, patients with Stage III‐IV periodontitis may require surgical treatment, which may include interventions aimed at regenerating the original tissue and its functionality.^[^ ^31^, ^32^, ^33^, ^34^, ^35^, ^36^, ^37^, ^38^, ^39^, ^40^, ^41^, ^42^ ^]^ However, periodontal regeneration is a complex process involving multiple tissue types.^[^ ^43^ ^]^ This requires differentiating several cell types in the proper location and a highly coordinated spatiotemporal healing response regulated by a biological signaling system.^[^ ^3^, ^5^, ^43^ ^]^ Periodontal regeneration is indicated for class II furcation defects and intrabony defects > 3 mm (**Figure** **1**) as it provides predictable and clinically relevant results, even though complete defect resolution is hardly ever achieved.^[^ ^43^, ^44^, ^45^, ^46^ ^]^ Comparatively, the regeneration of class III furcation defects currently remains challenging.^[^ ^45^, ^46^, ^47^, ^48^ ^]^ Apart from treating periodontitis, regenerative strategies are also employed for root coverage procedures to treat mid‐buccal gingival recessions, a highly prevalent condition associated with traumatic toothbrushing.^[^ ^49^, ^50^, ^51^, ^52^, ^53^ ^]^

This review aims to provide an overview of the current periodontal regenerative technologies used in clinical practice, discussing their composition, mechanism of action, and applications to shed light on the limitations in the field and suggest areas for future research.

## 2 Review of Regenerative Products in Periodontal Regeneration

### 2.1 Platelet Concentrates

**Figure 2:**
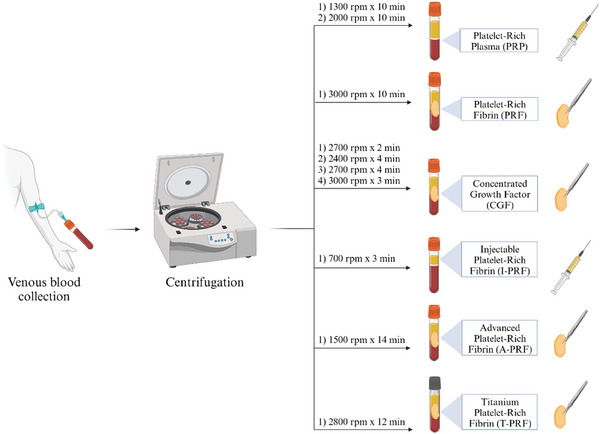
Preparation protocols of first‐, second‐, and third‐generation platelet concentrates.

Platelet concentrates (PC) are autologous bioactive products widely used in periodontal regeneration obtained from the centrifugation of venous blood (**Figure** **2**).^[^ ^54^ ^]^ They are composed of platelets, rich in growth factors, fibrin, which is the supporting matrix, and in some cases, a cellular component is included, mostly leukocytes.^[^ ^55^ ^]^ To have beneficial effects, the concentration of platelets must be ≈10^6^ per µL: lower concentrations are not optimal, while higher concentrations have an inhibitory effect.^[^ ^56^ ^]^ Platelets contain different concentrations of growth factors, namely primary fibroblast growth factor (FGF‐2 or bFGF), vascular endothelial growth factor (VEGF), insulin‐like growth factor‐1 (IGF‐1), transforming growth factor‐β (TGF‐β), and platelet‐derived growth factor‐BB (PDGF‐BB).^[^ ^55^, ^57^, ^58^, ^59^ ^]^ FGF‐2 shows angiogenic action and promotes the proliferation of marrow‐derived mesenchymal cells, with subsequent differentiation into osteoblasts.^[^ ^60^ ^]^ VEGF is pivotal in angiogenesis, controlling endothelial cell proliferation, migration, specialization, and survival.^[^ ^55^, ^61^ ^]^ IGF‐1 positively regulates the proliferation and differentiation of most cell types.^[^ ^55^, ^61^ ^]^ TGF‐β stimulates fibroblast chemotaxis and the production of collagen and fibronectin by cells, which improves fibrogenesis; moreover, TGF‐β promotes bone formation by stimulating osteoblast deposition while inhibiting osteoclast formation.^[^ ^55^, ^61^ ^]^ PDGF‐BB is the first growth factor present in wound healing.^[^ ^55^, ^61^ ^]^ It regulates angiogenesis, recruits mesenchymal stem cells, and favors the proliferation of periodontal ligament fibroblasts.^[^ ^56^, ^62^, ^63^ ^]^ Hence, the main advantage of platelet concentrates is the delivery of multiple growth factors that work synergistically at the wound site.^[^ ^55^ ^]^ Furthermore, in vitro studies showed that human platelets are a source of antimicrobial peptides, such as platelet factor 4 (PF‐4), connective tissue activating peptide 3 (CTAP‐3), thymosin β−4 (Tβ−4), platelet essential protein, fibrinopeptide A (FP‐A) and B (FP‐B).^[^ ^64^ ^]^ However, Yang et al. highlighted how PCs possess a bacteriostatic action rather than a bactericidal one.^[^ ^65^ ^]^

#### 2.1.1 First‐Generation Platelet Concentrates

Platelet‐rich plasma (PRP) is a first‐generation PC with a high platelet concentration but low natural fibrinogen.^[^ ^66^ ^]^ It is obtained through a two‐step centrifugation procedure of venous blood that requires the use of xenogenic thrombin and anticoagulant: the first step is low‐revolution spin (1300 rpm for 10 min) while the second is high‐revolution spin (2000 rpm for 10 min).^[^ ^54^, ^59^, ^66^ ^]^ PRP releases most growth factors in the first hours and dissolves entirely after 3 days.^[^ ^57^, ^59^ ^]^ It has been demonstrated in vitro to aid oral osteoblasts and gingival and periodontal ligament fibroblasts, thus promoting periodontal regeneration.^[^ ^55^ ^]^ PRP can also be obtained in a leukocyte‐rich form, referred to as L‐PRP: the presence of immune cells could further enhance the antimicrobial properties, even though the major effect is believed to be ascribable to platelets.^[^ ^55^, ^65^ ^]^ PRP has been used clinically for periodontal regeneration, demonstrating improved clinical outcomes.^[^ ^67^, ^68^, ^69^, ^70^ ^]^ Specifically, PRP combined with a β‐tricalcium phosphate graft (β‐TCP + PRP, Test group) showed significantly better performances than β‐TCP alone (Control group) in intra‐bony defects after 36 weeks (Probing depth reduction – Control: 2.20 ± 0.21 mm, Test: 2.80 ± 0.25 mm; Clinical Attachment Level Gain – Control: 1.10 ± 0.24 mm, Test: 1.80 ± 0.31 mm).^[^ ^68^ ^]^ The adjunctive benefit can be ascribed to both the release of growth factors and PRP's ability to stabilize the graft with its sticky consistency.^[^ ^68^ ^]^

Nonetheless, the manufacturing process of PRP is costly and involves biochemical modification, leading to various drawbacks.^[^ ^71^ ^]^ These include potential immune reactions from xenogenic thrombin and interference of anticoagulants with angiogenic and regenerative responses.^[^ ^58^, ^72^ ^]^ Additionally, PRP should be prepared and used within a 4‐hour timeframe, and it offers only a short‐term effect.^[^ ^73^ ^]^ Consequently, PRP has been less frequently employed in recent times.

#### 2.1.2 Second‐Generation Platelet Concentrates

To overcome the limitations of PRP, a second‐generation PC, platelet‐rich fibrin (PRF), was introduced. Unlike PRP, PRF is obtained with a single high‐revolution spin centrifugation step (3000 rpm for 10 min) of venous blood in a glass tube without biochemical modification.^[^ ^66^, ^74^ ^]^ The main biological difference between PRP and PRF is polymerization. While in PRP this is artificial, and there is extrinsic growth factor enmeshment, in PRF the polymerization is natural with intrinsic growth factor enmeshment.^[^ ^55^ ^]^ In PRF, this is possible thanks to the fibrinogen it contains, which is converted into fibrin under the influence of the physiologically available thrombin.^[^ ^57^ ^]^ Moreover, PRF releases growth factors over a more extended period (10 days) than PRP and has immunological benefits, which explains why fewer post‐operative infections have been reported after using PRF compared to PRP.^[^ ^57^, ^59^ ^]^ Similarly to PRP, PRF can be found in a leukocyte‐rich form (L‐PRF), obtained by centrifugation of venous blood at 2700 rpm for 12 min.^[^ ^66^, ^71^, ^75^ ^]^ Clinically, PRF and L‐PRF have been extensively applied to periodontal regeneration, alone or in combination with other regenerative materials, with PRF particularly favoring soft tissue over hard tissue regeneration.^[^ ^70^, ^72^, ^76^, ^77^, ^78^, ^79^, ^80^, ^81^, ^82^, ^83^, ^84^, ^85^, ^86^, ^87^, ^88^, ^89^, ^90^, ^91^, ^92^, ^93^, ^94^, ^95^, ^96^, ^97^, ^98^, ^99^, ^100^, ^101^, ^102^, ^103^, ^104^, ^105^, ^106^, ^107^ ^]^ In a randomized clinical trial on 3‐wall intra‐bony defects, Sharma et al. found that PRF combined with open flap debridement (OFD) yields significantly better results than OFD alone for probing pocket depth reduction (PRF + OFD: 4.55 ± 1.87 mm; OFD: 3.21 ± 1.64 mm), clinical attachment level gain (PRF + OFD: 3.31 ± 1.76 mm; OFD: 2.77 ± 1.44 mm) and percentage bone fill (PRF + OFD: 48.26% ± 5.72%; OFD: 1.80% ± 1.56%).^[^ ^101^ ^]^ The morphology of the defects involved has also aided the benefits of PRF because the walls provide space maintenance. Further histological data are needed to confirm the ability of PRF to promote bone regeneration.^[^ ^59^, ^72^ ^]^

#### 2.1.3 Third‐Generation Platelet Concentrates

It has been noted that the centrifugal force directly influences the PC composition: as the centrifugation speed decreases, growth factors and leukocyte release from the PRF clot increases.^[^ ^59^, ^75^, ^108^, ^109^ ^]^ This led to the recently developed third‐generation platelet concentrates, namely concentrated growth factor (CGF), injectable platelet‐rich fibrin (I‐PRF), advanced platelet‐rich fibrin (A‐PRF), and titanium platelet‐rich fibrin (T‐PRF).

CGF is manufactured in a glass‐coated plastic tube through a centrifugation program involving 30 seconds of acceleration followed by 2700 rpm for 2 min, 2400 rpm for 4 min, 2700 rpm for 4 min, 3000 rpm for 3 min, and 36 s of deceleration.^[^ ^59^, ^110^, ^111^ ^]^ Concerning PRF, CGF has a denser and richer fibrin matrix, which permits a slower release of growth factors.^[^ ^59^, ^110^ ^]^ Additionally, CGF can release chemokines responsible for cell recruitment.^[^ ^110^ ^]^

I‐PRF is obtained from the centrifugation of venous blood in plastic tubes at 700 rpm for three minutes.^[^ ^108^ ^]^ With this preparation protocol, there are more regenerative cells with a greater concentration of growth factors, but the main component is fibronectin, which promotes cellular growth when applied to root surfaces.^[^ ^57^, ^71^, ^108^ ^]^ The great advantage of I‐PRF is its injectability, which is given by the presence of fibrin‐generating small clots with dynamic gel properties.^[^ ^57^ ^]^ I‐PRF promotes an increased rate of migration of fibroblasts and the expression of TGF‐β and PDGF‐BB.^[^ ^59^, ^108^ ^]^ It affects the osteoblastic behavior and enhances periodontal regeneration in the treatment of gingival recessions, intra‐bony defects, and furcation defects.^[^ ^58^, ^71^, ^108^ ^]^

A‐PRF requires centrifugation of venous blood in a glass tube at 1500 rpm for 14 min and has a more porous‐like structure with bigger interfibrous space than PRF.^[^ ^108^ ^]^ Additionally, A‐PRF increases the expression of VEGF, which is advantageous for the angiogenesis of the gingiva.^[^ ^71^, ^108^ ^]^

T‐PRF is obtained by centrifuging venous blood in medical‐grade titanium tubes at 2800 rpm for 12 min.^[^ ^108^ ^]^ Titanium has a higher hemocompatibility than glass, which leads to a firmer, thicker, and more densely woven fibrin matrix than PRF.^[^ ^108^ ^]^ A strong fibrin structure is essential to delay the resorption time of fibrin during wound healing, thus increasing the delivery time of growth factors.^[^ ^71^, ^108^ ^]^ Moreover, T‐PRF releases a higher concentration of VEGF, PDGF‐BB, TGF‐β and IGF‐1.^[^ ^108^ ^]^

Clinically, CGF is mainly used for hard tissue regeneration.^[^ ^59^ ^]^ Clinical trials indicate its superior efficacy to surgical procedures alone for alveolar ridge preservation and intra‐bony defects.^[^ ^112^, ^113^ ^]^ CGF has also been coupled with bone grafts, although the combination did not yield further improvement concerning the bone graft alone.^[^ ^113^, ^114^ ^]^ However, the benefits of this combination are related to the number of residual bony walls: the higher the number, the easier it is for CGF to promote cell proliferation and differentiation required during wound healing.^[^ ^113^ ^]^

Yet few clinical studies on I‐PRF, A‐PRF, and T‐PRF in periodontal regeneration are available, and they failed to demonstrate superiority over the control groups.^[^ ^115^, ^116^, ^117^ ^]^ Therefore, further research and long‐term studies are needed to verify their beneficial effect.

### 2.2 Bone Grafts

Bone grafts are widely used in periodontal regeneration as structural frameworks for space maintenance with osteoconductive features that promote bone healing.^[^ ^118^ ^]^ To achieve bone regeneration, bone grafts must be integrated into the defect site, including inflammation, revascularization, osteoinduction, osteoconduction, and remodeling.^[^ ^119^ ^]^ For this purpose, bone grafts must mechanically support cells' adhesion, growth, and proliferation.^[^ ^119^ ^]^ This is achieved with correct pore morphology, interconnectivity, surface structure, and surface area to volume ratio together with mechanical and chemical properties.^[^ ^120^ ^]^ Examples of commercial bone grafts clinically used for periodontal regeneration are listed in **Table** **1**. Depending on the origin, bone grafts can be classified as autografts, allografts, xenografts, and alloplasts (synthetic).^[^ ^118^, ^119^, ^120^ ^]^

**Table 1:** Commercial bone grafts used clinically for periodontal regeneration.

Product	Manufacturer	Description	Category	Clinical studies
Bio‐Oss	Geistlich, Switzerland	Deproteinized bovine heterologous bone, only mineral component with Ca/P ratio equal to 2.03	Bovine xenograft	[67, 77, 84, 91, 102, 113, 129, 130, 131, 132, 133, 134, 135, 136, 137, 138, 139]
Bio‐Oss Collagen	Geistlich, Switzerland	90% Geistlich Bio‐Oss micro granules and 10% highly purified porcine collagen	Bovine xenograft	[140]
Tutodent Chips	Tutogen Medical GmbH, Germany	Particulate graft from cancellous bovine bone	Bovine xenograft	[83, 141]
Cerabone	Botiss Biomaterials GmbH, Germany	Pure bone mineral of bovine origin composed of hydroxyapatite	Bovine xenograft	[142, 143, 144, 145]
ABM/PepGen P‐15	Dentsply Sirona, USA	Anorganic bovine‐derived bone matrix combined with the synthetic cell‐binding peptide P‐15	Bovine xenograft	[146]
Bio‐Gen graft	Bioteck, Italy	Mixture of cancellous and cortical bone granules of equine origin	Equine xenograft	[147]
THE Graft	Purgo Biologics, Korea	Porous inorganic bone material of porcine origin	Porcine xenograft	[136, 148, 149]
OraGRAFT	LifeNet Health, USA	Demineralized freeze‐dried bone allograft combining 70% mineralized ground cortical with 30% demineralized ground cortical	Allograft	[90, 150]
AlloGro	AlloSource, USA	Decalcified freeze‐dried bone allograft with particle size 125–710 µm, made of human cortical powder	Allograft	[151]
NovaBone Dental Morsels	NovaBone Products, USA	Calcium phosphosilicate synthetic material composed of SiO_2_, Ca, Na_2_O, H and P	Synthetic graft	[79]
Perioglas	NovaBone Products, USA	Granulated form of Bioglass 45S5 consisting of 45.0%w SiO_2_, 6.0%w P_2_O_5_, and 24.5%w CaO and Na_2_O, respectively	Synthetic graft	[152, 153, 154]
Bone Ceramic	Straumann AG, Switzerland	Hydroxyapatite (60%) and β‐tricalcium phosphate (40%)	Synthetic graft	[155, 156]
ProRoot MTA	Dentsply Maillefer, Switzerland	Oxide‐based cement (60–90%) and bismuth trioxide (10–40%)	Synthetic graft	[69]
OsteoGen	Federico Maggi S.r.l. Unipersonale, Italy	Synthetic, granular, osteoconductive, and non‐ceramic crystalline forms of hydroxyapatite	Synthetic graft	[157]
SyboGraf	Eucare Pharmaceuticals, India	Synthetic nanocrystalline hydroxyapatite in powder/granule form	Synthetic graft	[93, 98, 158, 159]
Ceros TCP granules	Mathys European Orthopaedics, Switzerland	Synthetic, osteoconductive bone substitute consisting of β‐tricalcium phosphate	Synthetic graft	[160]
Gem 21S	Osteohealth, USA	β‐tricalcium phosphate and recombinant human platelet‐derived growth factor (rhPDGF‐BB)	Synthetic graft	[161]
MD05	Scil Technology and Ceraver Osteal, France	Particulate porous β‐tricalcium phosphate and recombinant human growth/differentiation factor‐5 (rhGDF‐5)	Synthetic graft	[162]
Frios Algipore	Friadent GmbH, Germany	Biological hydroxyapatite derived from porous‐apatite of lime‐encrusted ocean algae	Synthetic graft	[163]
Cytrans Granules	GC Corporation, Japan	Carbonated apatite granules	Synthetic graft	[164]
Fisiograft	Ghimsa S.p.A., Italy	Copolymer of polylactic and polyglycolic acids in a 50:50 ratio	Synthetic graft	[165]

#### 2.2.1 Autogenous Bone Grafts

Autogenous bone grafts (ABG) are obtained from the patients themselves and are considered the gold standard for bone defect repair because they are not immunogenic and show osteogenic, osteoconductive, and osteoinductive properties.^[^ ^118^, ^119^, ^120^, ^121^ ^]^ However, autografting requires additional surgery, and there can be issues with patient morbidity and a limited amount of material available.^[^ ^118^, ^119^, ^120^, ^121^ ^]^ Furthermore, its fast resorption, leading to a subsequent loss of the space‐maintaining effect, and concerns related to the possibility of external root resorption have been described.^[^ ^122^ ^]^ Yet autografts have found clinical applications.^[^ ^82^, ^104^, ^123^, ^124^ ^]^ A comparative clinical trial using either PRF or ABG to treat 3‐wall intra‐bony defects demonstrated statistically significant improvements in probing depth (PRF: 3.20 ± 0.919 mm, ABG: 2.60 ± 0.843 mm) and radiographic defect depth (PRF: 4.6 ± 0.7 mm, ABG: 4.1 ± 0.4 mm) after nine months.^[^ ^82^ ^]^ It should be noted that only the radiographic bone fill was significantly different in the two groups, with better results for the ABG group, probably due to the volume‐filling capability of the ABG.^[^ ^82^ ^]^

#### 2.2.2 Allografts

Allografts, sourced from another human, prevent secondary surgery in the patient.^[^ ^118^, ^119^, ^120^, ^121^ ^]^ Nonetheless, it should be considered that the tissue is not standardized because of the differences in age, gender, and medical history of the donors; moreover, the availability of the material is not ensured.^[^ ^118^, ^119^, ^120^, ^121^ ^]^ It is fundamental to adequately reduce the risk of rejection and disease transmission of allografts.^[^ ^125^ ^]^ Indeed, recently, using an allograft in the USA led to disseminated tuberculosis to recipients, who experienced substantial morbidity and mortality.^[^ ^125^ ^]^ Consequently, proper freeze‐drying and/or demineralization processes become of paramount importance.^[^ ^119^ ^]^ According to the treatment, allografts are referred to as freeze‐dried bone allografts (FDBA), demineralized freeze‐dried bone allografts (DFDBA), or demineralized bone matrices (DBM).^[^ ^121^ ^]^ The freeze‐drying process reduces the antigenicity, while the demineralization exposes the collagen and the bone morphogenetic proteins (BMP), and provides an osteoinductive potential.^[^ ^121^ ^]^

#### 2.2.3 Xenografts

Xenografts are animal‐derived bone grafts subjected to thermal and chemical treatments to limit immunogenicity.^[^ ^118^, ^119^, ^120^, ^121^ ^]^ Xenografts are primarily available, low‐cost, and have osteoconductive properties; they also give predictable clinical outcomes.^[^ ^119^, ^120^, ^121^ ^]^ Yet xenografts face issues such as lack of viable cells, immunogenicity, disease transmission, and variable resorption rate.^[^ ^119^, ^120^, ^121^ ^]^ Bovine bone is the most common as it is considered the closest to the human bone after autografts, but there are also bone grafts of porcine and equine origin.^[^ ^119^, ^120^ ^]^ Equine‐derived xenografts are remarkable for their ability to induce osteoblastic differentiation and angiogenesis while being remodeled by osteoclasts; porcine‐derived xenografts possess a structure similar to human bone.^[^ ^119^ ^]^

Bio‐Oss (Geistlich, Switzerland) is a bovine xenograft considered a leading product within the dental field, with extensive supporting clinical evidence (Table 1). In a randomized split‐mouth clinical trial, Lekovic and colleagues showed that Bio‐Oss can enhance the outcomes of periodontal regeneration in 2‐wall and 3‐wall intra‐bony defects when combined with PRF.^[^ ^91^ ^]^ This combination resulted in a better radiographic bone fill than PRF alone (Bio‐Oss + PRF: 4.06 ± 0.87 mm; PRF: 2.21 ± 0.68 mm) but also a more significant clinical attachment level gain (Bio‐Oss + PRF: 3.82 ± 0.78 mm; PRF: 2.24 ± 0.73 mm) at 6 months, which was statistically significant for both parameters.^[^ ^91^ ^]^ This suggested that Bio‐Oss guarantees space for tissue regeneration promoted by PRF, beyond osteoconductivity in 2‐ and 3‐wall intra‐bony defects. Whether this is also sufficient for 1‐wall intra‐bony defects is yet to be demonstrated.

#### 2.2.4 Alloplasts

Synthetic bone grafts are synthetic substitutes that can be based on polymeric or ceramic materials.^[^ ^119^, ^120^, ^121^, ^126^, ^127^ ^]^ The great advantage lies in the possibility of tuning the physicochemical properties of these materials.^[^ ^121^ ^]^

In periodontal regeneration, the commercial ceramic grafts are typically composed of bioactive glass or calcium phosphate, such as β‐tricalcium phosphate (β‐TCP), hydroxyapatite (HA), biphasic calcium phosphate (BCP).^[^ ^119^, ^121^, ^127^ ^]^ However, oxide‐based materials are also possible.^[^ ^120^ ^]^

Bioactive glasses consist of silica (SiO_2_), sodium oxide (Na_2_O), calcium oxide (CaO), and phosphorus pentoxide (P_2_O_5_), with particle size ranging from 90 to 710 µm to 300–355 µm.^[^ ^119^, ^121^, ^127^ ^]^ Being bioactive, this material can form a strong bond between the glass and the host bone through hydroxyapatite crystals.^[^ ^119^ ^]^ Moreover, bioactive glasses display exceptional biocompatibility, adaptability to clinical features, and antibacterial properties.^[^ ^128^ ^]^ Within this category, Perioglas (NovaBone Products, USA) is a well‐established product (Table 1).

Calcium phosphates are interesting materials as they affect the adsorption of extracellular matrix proteins on their surface, thus promoting cell adhesion and tissue formation.^[^ ^126^ ^]^ Specifically, calcium ions stimulate osteoblasts to promote bone regeneration while regulating bone resorption by osteoclasts.^[^ ^126^ ^]^ In addition, phosphate ions affect the differentiation and growth of osteoblasts and inhibit osteoclasts’ differentiation. β‐TCP resorbs quickly and exhibits low immunogenicity, but the mechanical properties are scarce.^[^ ^126^ ^]^ Comparatively, HA possesses a composition and structure similar to native bone minerals and resorbs more slowly.^[^ ^121^, ^126^ ^]^ To optimize the resorption rate, β‐TCP and HA can be mixed to obtain BCP, with a typical ratio of 40% β‐TCP and 60% HA.^[^ ^119^, ^126^ ^]^ This is the case for the commercial graft Bone Ceramic (Straumann AG, Switzerland) (Table 1).

Nonetheless, ceramic‐based materials tend to be brittle and do not have predictable absorption.^[^ ^120^ ^]^

Although less common due to scarce cellular adhesion, alteration of mechanical properties, and release of acidic degradation products, polymers can be used as bone grafts because of their biodegradability, biocompatibility, and tunable properties.^[^ ^120^, ^121^, ^126^ ^]^ This is the case for Fisiograft (Ghimsa S.p.A., Italy), a copolymer of polylactic and polyglycolic acids in a 50:50 ratio (Table 1).

### 2.3 Hydrogels

Several hydrogels for periodontal regeneration are available on the market (**Table** **2**). Hydrogels are water and blood‐absorbing polymer networks that can function as a temporary extracellular matrix (ECM) to facilitate tissue regeneration.^[^ ^166^ ^]^ The great advantage of hydrogels is their biocompatibility, fluidity, and injectability, as well as their ability to mimic the natural ECM with the correct tuning of their properties.^[^ ^167^, ^168^, ^169^ ^]^ Mainly, injectability is a highly desirable feature in periodontal regeneration, given the small size of the defects involved and the possibility of a minimally invasive clinical intervention.^[^ ^170^ ^]^ This can be achieved with physically crosslinked, shear‐thinning or thermosensitive hydrogels.^[^ ^171^ ^]^ To have functional periodontal regeneration, the gel's mechanical properties must remain consistent as bone regeneration can occur only if there is stable spacing for the bone to form.^[^ ^172^ ^]^

**Table 2:** Commercial hydrogels used clinically for periodontal regeneration.

Product	Manufacturer	Description	Clinical studies
Emdogain	Straumann AG, Switzerland	90% amelogenin plus other proteins such as ameloblastin, enamelin, and tuftelin in an aqueous solution of propylene glycol alginate	[85, 86, 123, 130, 131, 137, 142, 143, 148, 155, 156, 194, 195, 196, 222, 243, 244, 245, 246, 247, 248]
REGROTH Dental Kit	Kaken Pharmaceutical CO., Ltd, Japan	Recombinant human fibroblast growth factor (rhFGF‐2) in a hydroxypropyl cellulose carrier (3%)	[129, 139, 164, 201, 205, 206]
HyaDENT BG	Bioscience GmbH, Germany	Cross‐linked HA—HA gel composed of a mixture of cross‐linked (1.6%) and natural (0.2%) hyaluronic acid	[149, 222]
Gengigel	Ricerfarma, Italy	Water, 7.5% xylitol and excipients, 0.8% Sodium hyaluronate with pH of 6.5 ± 0.5 (20 °C)	[158, 227, 232, 233, 234]
Ossigel	Orquest, Inc., USA	4 mg mL^−1^ of Recombinant Human Fibroblast Growth Factor type 2 (rhFGF‐2) in a sodium hyaluronate (MW 1.39 MDa) carrier	[235]
Aminogam	Errekappa Euroterapici SpA, Italy	Glycine, leucine, proline, and lysine within a sodium hyaluronate carrier	[238, 241, 242]

#### 2.3.1 EMD

Enamel matrix derivative (EMD) is a purified acidic extract of enamel matrix proteins derived from unerupted porcine tooth buds, comprising 90% amelogenins and other proteins such as ameloblastin, tuftelin, enamelin, and amelotin.^[^ ^173^ ^]^ EMD mimics biological processes during periodontal tissue growth, where Hertwig's epithelial root sheath cells deposit enamel matrix proteins on the root surface, initiating cementogenesis.^[^ ^174^, ^175^ ^]^ Indeed, EMD precipitates and adsorbs on denuded and conditioned root surfaces and alveolar bony defects, forming a long‐lasting molecular scaffold complex that promotes periodontal regeneration.^[^ ^176^, ^177^ ^]^ EMD has been commonly used in periodontal regeneration for more than 25 years and is available commercially under the name Emdogain (Straumann AG, Switzerland), which is supplied in a sterile propylene glycol alginate (PGA) aqueous acidic solution.^[^ ^170^, ^173^, ^178^, ^179^ ^]^ PGA enhances EMD precipitation, exposing periodontal ligament cells to the re‐established protein aggregate, and allowing matrix‐cell interactions to occur.^[^ ^173^, ^180^ ^]^

Histological evidence supports EMD's ability to regenerate the various periodontal tissues.^[^ ^181^, ^182^, ^183^, ^184^ ^]^ However, its mechanism of action requires further clarification.^[^ ^178^, ^185^ ^]^ It has been shown that EMD significantly influences several cell types, mediating cell attachment, spreading, proliferation, differentiation, and survival.^[^ ^178^ ^]^ In vitro and in vivo studies demonstrate that EMD stimulates mesenchymal cell growth.^[^ ^173^ ^]^ Specifically, EMD favors mesenchymal cell growth over epithelial cells.^[^ ^174^, ^186^, ^187^, ^188^, ^189^ ^]^ Restricting epithelial cells is essential for regenerating all periodontal tissues since periodontal ligament cells, cementoblasts, and osteoblasts have a slower turnover rate.^[^ ^45^ ^]^ EMD also possesses angiogenic activity and favors wound healing.^[^ ^178^ ^]^ Overall, this can be associated with the direct matrix‐cell contact and with EMD stimulating the expression of transcription factors (Runx2 and Osterix), growth factors (TGF‐β, BMP, VEGF, FGF‐2, PDGF), cytokines (osteoprotegerin, IL‐6), and extracellular matrix constituents such as hyaluronan and proteoglycans.^[^ ^170^, ^173^, ^174^, ^176^, ^179^, ^189^, ^190^, ^191^, ^192^, ^193^ ^]^ These biological agents are involved in new alveolar bone, root cementum, and functional periodontal ligament formation.^[^ ^176^, ^178^, ^191^ ^]^

Clinical trials have confirmed the efficacy of Emdogain in improving the outcomes of periodontal regeneration.^[^ ^85^, ^86^, ^156^, ^194^, ^195^ ^]^ In a split‐mouth randomized study, Bhutda and Deo demonstrated the benefits of Emdogain combined with the conditioning agent Prefgel (neutral formulation of 24% ethylenediaminetetraacetic acid) and open flap debridement (OFD) when compared to Prefgel and OFD for the treatment of 2‐wall and 3‐wall intra‐bony defects.^[^ ^194^ ^]^ The clinical outcomes at five years and the radiographic outcomes at one year were significantly better when Emdogain was applied (probing pocket depth reduction: 3.84 ± 1.05 mm versus 1.92 ± 0.35 mm; clinical attachment level gain: 3.18 ± 0.87 mm versus 1.60 ± 0.54 mm; percentage radiographic bone fill: 66.66 ± 7.8% versus 31.71 ± 4.1%).^[^ ^194^ ^]^

Despite the supportive clinical evidence, some clinical trials indicate that Emdogain does not further improve the mean clinical and radiographic outcomes in periodontal regeneration.^[^ ^123^, ^137^, ^142^, ^143^, ^148^, ^155^, ^196^, ^197^ ^]^ Additionally, its fluid nature causes fast degradation and loss of mechanical properties.^[^ ^86^, ^198^ ^]^ This can lead to flap collapse and inhibit bone regeneration, especially in deep defects with fewer supportive bony walls.^[^ ^86^, ^178^ ^]^ Therefore, the combination of Emdogain with different space‐maintaining biomaterials has been proposed to address this issue.^[^ ^199^ ^]^ According to the systematic review conducted by Matarasso and colleagues, one combination that has displayed promising results is EMD combined with bone grafts.^[^ ^199^ ^]^ Compared to EMD alone, this combination has been found beneficial for both soft and hard tissue metrics.^[^ ^199^ ^]^

#### 2.3.2 REGROTH Dental Kit

REGROTH Dental Kit (Kaken Pharmaceutical Co., Ltd, Japan) is a hydrogel formulation consisting of 0.3% recombinant human FGF‐2 (rhFGF‐2) in a 3% hydroxypropyl cellulose (HPC) carrier.^[^ ^164^, ^200^ ^]^ It is supplied in two separate cartridges, with lyophilized rhFGF‐2 being mixed with the HPC carrier before use.^[^ ^164^, ^200^ ^]^ The active ingredient, rhFGF‐2, has a potent angiogenic action and promotes the proliferation of undifferentiated mesenchymal cells while keeping their pluripotency.^[^ ^60^, ^164^, ^200^, ^201^ ^]^ In addition, it stimulates human periodontal ligament cells to produce a wide range of extracellular matrix molecules and VEGF.^[^ ^200^ ^]^ Hydroxypropyl cellulose is a water‐soluble polymer that acts as a hydrogel scaffold.^[^ ^202^ ^]^ Histological studies on animals have shown functional periodontal ligament formation with new cementum deposition and bone formation after applying rhFGF‐2.^[^ ^203^, ^204^ ^]^ Clinical trials have extensively proven the efficacy of REGROTH Dental Kit, improving both clinical and radiographic outcomes.^[^ ^129^, ^139^, ^164^, ^201^, ^205^, ^206^ ^]^ Interestingly, the efficacy of rhFGF‐2 was not affected by patients’ age, sex, type of tooth, and classification of bone defects.^[^ ^206^ ^]^ Moreover, in a randomized controlled clinical study on 2‐ and 3‐wall intra‐bony defects, REGROTH Dental Kit (Group 1) was found to be superior to Emdogain (Group 2) and OFD (Group 3) in radiographic bone fill after 36 weeks (Group 1: 34.37 ± 24.42%, Group 2: 23.29 ± 25.11%, Group 3: 13.30 ± 20.60%).^[^ ^206^ ^]^ Nevertheless, while the clinical attachment level gain was higher when applying REGORTH Dental Kit or Emdogain than OFD alone, there was no statistically significant difference between Group 1 and Group 2 (Group 1: 2.70 ± 1.29 mm, Group 2: 2.30 ± 1.51 mm, Group 3: 1.70 ± 1.39 mm).^[^ ^206^ ^]^

**Figure 3:**
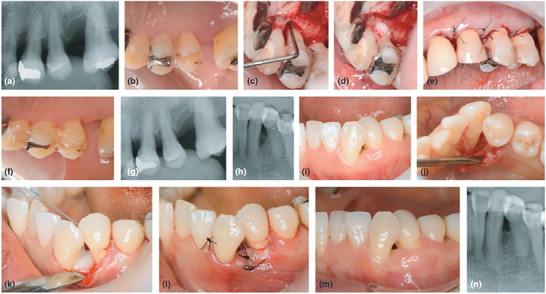
Surgical procedures and outcomes. a–g) Test group (REGROTH Dental Kit + Bio‐Oss). a) Preoperative radiograph. b) Baseline clinical view. c) Intra‐operative view. d) REGROTH Dental Kit + Bio‐Oss application. e) Suturing. f) 6‐month follow‐up view. g) 6‐month follow‐up radiograph. h–n) Control group (REGROTH Dental Kit). h) Preoperative radiograph. i) Baseline clinical view. (j) Intra‐operative view. k) REGROTH Dental Kit application. l) Suturing. m) 6‐month follow‐up view. n) 6‐month follow‐up radiograph. Reproduced with permission.^[^ ^129^ ^]^ Copyright 2019, John Wiley & Sons Ltd.

REGROTH Dental Kit's regenerative potential might be limited for severe intra‐bony defects.^[^ ^164^, ^200^ ^]^ In these cases, the combination with bone grafts has been investigated in clinical trials, showing promising clinical and radiographic results.^[^ ^129^, ^164^ ^]^ Particularly, in a randomized clinical trial Saito et al. tested REGROTH Dental Kit combined with a xenogenic bone graft (Bio‐Oss, Geistlich, Switzerland) against REGROTH Dental Kit alone for the treatment of intra‐bony defects, revealing a significant improvement in clinical attachment level gain from baseline for both treatments (REGROTH Dental Kit + Bio‐Oss: 3.16 ± 1.45 mm; REGROTH Dental Kit: 2.77 ± 1.15 mm) and a statistically significant greater percentage bone fill in the bone graft group (REGROTH Dent al Kit + Bio‐Oss: 47.2%; REGROTH Dental Kit: 29.3%) after 6 months (**Figure** **3**).^[^ ^129^ ^]^ It is noteworthy that, for both therapies, there was a significant improvement between baseline and 3 months, but not between 3 and 6 months, except for the percentage of bone fill. This can suggest that the benefit from rhFGF‐2 can primarily be observed in the short term through accelerated tissue regeneration. After 4 years, the improvements in clinical and radiographic outcomes were maintained.^[^ ^139^ ^]^ Specifically, there were no statistically significant differences in clinical attachment level between the groups, but the bone graft group showed significantly improved radiographic bone fill, plausibly because of its volume‐filling capabilities.^[^ ^139^ ^]^ However, the graft material has not been subtracted in the analysis.

The available clinical trials have all demonstrated the safety of REGROTH Dental Kit.^[^ ^129^, ^139^, ^164^, ^201^, ^205^, ^206^ ^]^ However, care must be taken by clinicians since there is the possibility that applying the drug to a site containing tumour cells may promote their proliferation.^[^ ^200^ ^]^

#### 2.3.3 Hyaluronan‐Based Hydrogels

Many of the gels available on the market are based on hyaluronan, which can be either hyaluronic acid or a salt, such as sodium hyaluronate.^[^ ^207^ ^]^ The properties of hyaluronan can vary depending on the molecular weight and degree of crosslinking.^[^ ^208^, ^209^ ^]^ However, hyaluronan is generally known for its hydrophilic, non‐adhesive, biodegradable, resorbable, anti‐inflammatory, and immunosuppressive characteristics.^[^ ^208^ ^]^ Moreover, hyaluronan has been found to promote bone regeneration and play a role in angiogenesis and wound healing.^[^ ^208^, ^209^, ^210^ ^]^ Additionally, periodontal ligament (PDL) cells express the transmembrane glycoprotein CD44, which is present during tooth development and serves as the principal receptor for hyaluronic acid.^[^ ^211^, ^212^, ^213^ ^]^ CD44 upregulates PDL cells’ proliferation and mineralization, and studies indicate that CD44 is involved in their contractility and migration in response to hyaluronic acid.^[^ ^213^, ^214^ ^]^ Also, hyaluronic acid is present in the ECM of periodontal tissues, with a higher concentration in the soft tissues than in the hard tissues.^[^ ^210^, ^215^ ^]^ These qualities make hyaluronan‐based gels highly similar to the ECM.^[^ ^216^ ^]^ Scientific evidence supports the topical application of these gels for treating gingivitis, and for non‐surgical and surgical periodontitis treatment.^[^ ^207^, ^210^, ^217^, ^218^, ^219^ ^]^

##### HyaDENT BG

**Figure 4:**
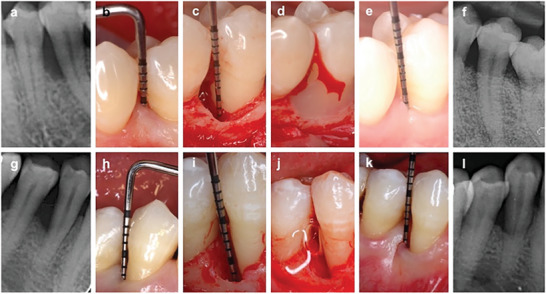
a–f) Control group (Emdogain). (a) Baseline radiograph view. (b) Baseline clinical view. (c) Intraoperative view. (d) Emdogain application. (e) 24‐month follow‐up clinical view. (f) 24‐month follow‐up radiographic view. g–l) Test group (HyaDENT BG). (g) Baseline radiographic view. (h) Baseline clinical view. (i) Intraoperative view. (j) HyaDENT BG application. (k) 24‐month follow‐up clinical view. (l) 24‐month follow‐up radiographic view. Reproduced under terms of the CC‐BY 4.0 license.^[^ ^222^ ^]^ Copyright 2021, The Authors, published by Springer Nature.

HyaDENT BG (Bioscience GmbH, Germany) is a hydrogel formulation containing biotechnologically produced synthetic hyaluronic acid (MW = 1000 kDa) cross‐linked with butanediol diglycidyl ether (BDDE) and non‐crosslinked hyaluronic acid (MW = 2500 kDa) in a ratio 8:1 to a total concentration of 18 mg mL^−1^.^[^ ^149^, ^220^, ^221^ ^]^ The adjunct of cross‐linked hyaluronic acid slows down the resorption rate of the gel, making it suitable for clinical use.^[^ ^220^ ^]^ In vitro studies have demonstrated the ability of hyaDENT BG to expand osteoprogenitor cells while keeping their stemness and enhancing the proliferative, migratory, and wound‐healing properties of gingival fibroblasts.^[^ ^220^, ^221^ ^]^ This makes it a beneficial approach to regenerative periodontal surgery. Clinical trials involving hyaDENT BG have been conducted.^[^ ^149^, ^222^ ^]^ In a randomized clinical trial comparing hyaDENT BG to Emdogain in treating intra‐bony defects, Pilloni et al. observed statistically significant improvements in clinical parameters at 24 months with respect to baseline for both treatments (**Figure** **4**).^[^ ^222^ ^]^ However, Emdogain yielded superior results in probing pocket depth reduction (Emdogain: 4.5 ± 1.0 mm; hyaDENT BG: 3.3 ± 0.7 mm) and clinical attachment level gain (Emdogain: 2.94 ± 1.12 mm; hyaDENT BG:2.19 ± 1.11 mm), despite statistical significance was only present for the former.^[^ ^222^ ^]^ Yet the adsorption of EMD onto the root surfaces might be negatively affected by the surrounding blood contamination.^[^ ^223^ ^]^ As such, hyaluronic acid combined with surgical procedures may still represent a valuable alternative to Emdogain. In another trial on intra‐bony defects, Božić and colleagues demonstrated that the combination of hyaDENT BG with a porcine xenograft (THE Graft, Purgo Biologics Inc., Korea) resulted in significant clinical attachment level gain and probing depth reduction.^[^ ^149^ ^]^ Moreover, Guldener and Lanzrein utilized hyaDENT BG combined with a subepithelial connective tissue graft to treat gingival recession, achieving positive results in terms of root coverage.^[^ ^224^, ^225^ ^]^ However, the absence of control groups in the studies conducted by Božić, Guldener, and Lanzrein limits the comprehensive analysis of their findings.^[^ ^149^, ^224^, ^225^ ^]^

##### Gengigel

Gengigel (Ricerfarma S.r.l., Milan, Italy) is a hydrogel available in two different formulations, namely Gengigel oral gel (0.2% sodium hyaluronate, 7.5% xylitol, excipients) and Gengigel Prof syringes (0.8% sodium hyaluronate, 7.5% xylitol, excipients).^[^ ^226^, ^227^ ^]^ Hyaluronan is derived from *S. equi* and has a molecular weight ranging from 1000 kDa to 1890 kDa.^[^ ^215^ ^]^ Adding xylitol to the formulation might contribute to the gel's efficacy. Indeed, xylitol cannot be metabolized by the bacteria in the oral cavity, which limits their growth.^[^ ^228^ ^]^ Moreover, xylitol possesses an antiplaque effect, favors enamel remineralization, and reduces gingival inflammation.^[^ ^228^ ^]^

Gengigel oral gel is primarily adopted for gingivitis and non‐surgical periodontal therapy combined with sub‐gingival instrumentation and has shown benefits for soft tissue metrics.^[^ ^226^, ^229^, ^230^ ^]^

Gengigel Prof syringes, while have also been used in non‐surgical periodontal therapy, are mainly applied during surgical periodontal therapy.^[^ ^231^ ^]^ Clinical trials support their efficacy in improving both clinical and radiographic outcomes.^[^ ^158^, ^227^, ^232^, ^233^, ^234^ ^]^ Gupta et al. showed that Gengigel Prof syringes used within an OFD promote hard tissue metrics in treating class II furcation defects compared to OFD alone.^[^ ^234^ ^]^ However, it is difficult to ascertain the individual contributions of xylitol and hyaluronan to the regeneration process. Similarly, in a randomized clinical trial, Mamajiwala and colleagues investigated the treatment of 2‐wall and 3‐wall intra‐bony defects in chronic periodontitis patients using OFD with either Gengigel Prof syringes or a placebo gel containing carboxymethylcellulose, xylitol, sodium chloride, and water.^[^ ^227^ ^]^ After 12 months, the authors found statistically significant improvements in clinical attachment level gain (5.1 ± 0.7 mm vs 3.9 ± 0.9 mm), probing pocket depth reduction (5.4 ± 1.1 mm vs 4.2 ± 0.6 mm), and radiographic bone fill (5.67 ± 2.01 mm vs 4.49 ± 1.78 mm) when Gengigel Prof syringes were applied.^[^ ^227^ ^]^ The difference was even higher if 3‐wall intra‐bony defects only were considered, which can be simply attributed to the defect morphology. The placebo gel also contains xylitol, so these effects can be attributed to hyaluronan. These findings also align with those reported by Fawzy El‐Sayed and colleagues.^[^ ^232^ ^]^ Additionally, the clinical studies conducted by Selvaprakash and Bhowmik revealed that adding Gengigel Prof syringes to bone graft materials further enhances the clinical and radiographic outcomes, possibly because of better space‐maintaining properties.^[^ ^158^, ^233^ ^]^

##### Ossigel

Ossigel (Orquest, Inc., Mountain View, CA, USA) is a hydrogel containing 4 mg/ml of recombinant human fibroblast growth factor (rhFGF‐2) in a sodium hyaluronate (MW = 1390 kDa) carrier.^[^ ^235^ ^]^ Previous clinical trials by Kitamura have demonstrated the effectiveness of rhFGF‐2 in periodontal regeneration using REGROTH dental kit, which has a hydroxypropyl cellulose carrier.^[^ ^201^, ^205^ ^]^ In contrast, Ossigel utilizes a hyaluronan carrier due to the significant biological interactions and potential synergistic effects between hyaluronan and FGF‐2.^[^ ^235^ ^]^ Studies have shown that FGF‐2 can stimulate hyaluronan production in periodontal ligament cells.^[^ ^236^ ^]^ Moreover, research on baboons has revealed that this gel formulation promotes bone formation during fracture healing.^[^ ^237^ ^]^ To evaluate the efficacy of Ossigel in periodontal regeneration, a randomized clinical trial compared its use in combination with open debridement and papilla preservation flaps against the surgical procedure alone for treating intra‐bony defects.^[^ ^235^ ^]^ The group receiving Ossigel, exhibited significantly better regenerative outcomes at 12 months, including a higher gain in clinical attachment level (4.8 ± 0.2 mm vs 2.2 ± 0.5 mm) and reduction in probing pocket depth (5.5 ± 1.4 mm vs 2.9 ± 0.9 mm).^[^ ^235^ ^]^ However, whether the observed effect was to be primarily attributed to hyaluronan or rhFGF‐2 cannot be definitively determined. Considering that FGF‐2 has a strong pharmacological action and is effective in periodontal regeneration, it is plausible that its presence in Ossigel mainly contributed to the clinical outcomes.^[^ ^60^, ^201^, ^205^ ^]^ Further research could provide additional insights into the regenerative capabilities of this formulation in periodontology.

##### Aminogam

Aminogam Gingival Gel (Errekappa Euroterapici SpA, Milan, Italy) is a hydrogel formulation that contains amino acids, specifically glycine, leucine, proline, and lysine, within a sodium hyaluronate carrier.^[^ ^238^, ^239^ ^]^ Aminogam has been found to have multiple beneficial effects on wound healing, accelerating the healing process, promoting the proliferation of fibroblasts, stimulating angiogenesis and the release of VEGF by fibroblasts, and enhancing the expression of TGF‐β and IL‐6.^[^ ^239^, ^240^, ^241^ ^]^ Amino acids play a significant role in wound healing by contributing to collagenogenesis and forming the extracellular matrix.^[^ ^239^ ^]^ Additionally, hyaluronan and its degradation products are known for their anti‐inflammatory effects and their support of angiogenesis.^[^ ^239^, ^240^ ^]^ A controlled clinical study by Cosola et al. demonstrated that Aminogam can reduce swelling and pain in the soft tissues after tooth extraction.^[^ ^242^ ^]^ In a randomized split‐mouth clinical trial conducted by Bevilacqua et al., eleven patients with chronic periodontitis were treated with ultrasonic mechanical instrumentation either alone (Control group) or in combination with the subgingival application of Aminogam (Test group).^[^ ^238^ ^]^ After 90 days, the probing pocket depth reduction was significantly greater in the test group (6.14 ± 0.44 mm to 4.64 ± 0.54 mm) than in the control group (from 6.36 ± 0.50 mm to 5.36 ± 0.57 mm), suggesting that Aminogam is effective in soft tissue regeneration.^[^ ^238^ ^]^ Nonetheless, it should be considered that the clinical literature about Aminogam is scarce.

### 2.4 Bone Putties

Hydrogels typically have scarce space‐maintaining properties, which can limit the bone regeneration of periodontal defects.^[^ ^172^ ^]^ Therefore, they are often coupled with bone grafts in the clinics.^[^ ^249^ ^]^ An alternative approach would be to have regenerative products in putty form, namely particulate bone grafts in an absorbable binder.^[^ ^106^, ^107^, ^250^ ^]^ Indeed, the particle phase augments the graft solidity and the physical properties, thus facilitating the placement of the product into the defect and allowing it to remain firm.^[^ ^81^, ^163^, ^250^ ^]^ Furthermore, the voids between the particles permit fast vascularization and bone ingrowth.^[^ ^163^ ^]^ Commercial putties used for periodontal regeneration are listed in **Table** **3**.

**Table 3:** Commercial bone putties used clinically in periodontal regeneration.

Product	Manufacturer	Description	Clinical studies
NovaBone Putty	NovaBone Products, USA	Bioactive Glass (calcium phosphosilicate synthetic material composed of SiO_2_, Ca, Na_2_O, H, and P) plus polyethylene glycol and glycerine as additives	[81, 106, 107, 154, 159, 163, 251]
C‐Blast Putty	Citagenix Inc, Canada	Osteoinductive Demineralized Bone Matrix with osteoconductive cancellous bone particles in a carboxymethyl cellulose carrier	[253]
MaxResorb Inject	Botiss Biomaterials GmbH, Germany	Water‐based paste containing 16.5% of nano‐hydroxyapatite particles and biphasic granules (HA/β‐TCP in a 60:40 ratio)	[138, 145]
DBX Putty	MTF Biologics, USA	Granulated allogenic cortical bone (31% wt.) in a 4% sodium hyaluronate carrier with a pH of 7.2	[255]
Ostim	Heraeus Kulzer, Germany	35% nanocrystalline hydroxyapatite particles in aqueous dispersion	[243, 262, 265, 266]
MinerOss Putty	Biohorizons Implant Systems Inc., USA	Freeze‐dried allogenic mineralized cortical and cancellous chips along with demineralized cortical fibers in a 50:50 ratio, encapsulated within a natural allograft collagen carrier.	–^a^ ^)^
RegenerOss Allograft Putty Plus	ZimVie, USA	Cortical demineralized bone matrix (28%) and cancellous mineralized bone chips (20%) within a lecithin carrier	–^a^ ^)^

^a)^ Lack of clinical studies.

#### 2.4.1 NovaBone Putty

NovaBone Putty (NovaBone Products, USA) is a regenerative product composed of bioactive glass with additives such as polyethylene glycol and glycerine exhibiting osteostimulatory and osteoconductive properties.^[^ ^81^, ^106^, ^107^, ^154^, ^159^, ^163^, ^251^ ^]^ The bioactive glass is present in two different dimensional phases, namely 90–710 µm and 32–125 µm, and is composed only of elements that are present naturally in native bone, i.e., calcium, phosphorous, sodium, silicon, and oxygen.^[^ ^81^, ^106^, ^163^ ^]^ Bioactive glass can release biologically active soluble Si^4+^ and Ca^2+^ ions, which stimulate bone growth and can enhance the secretion of VEGF while retarding the growth of epithelial tissue.^[^ ^107^, ^163^, ^252^ ^]^ The additives improve the handling and efficacy in clinical applications.^[^ ^81^, ^107^, ^154^, ^251^ ^]^

Clinical trials involving NovaBone Putty have demonstrated its regenerative capabilities in soft and hard tissue.^[^ ^81^, ^106^, ^107^, ^154^, ^159^, ^163^, ^251^ ^]^ However, one study conducted by Asmita and colleagues suggests that NovaBone Putty produces statistically similar results for the particulate form Perioglas (NovaBone Products, USA) when used for class II furcation defects.^[^ ^154^ ^]^ Specifically, the mean resolution in vertical defects at 6 months was 50.48 ± 16.47% for Perioglas and 43.48 ± 9.33% for NovaBone Putty.^[^ ^154^ ^]^ On the other hand, Bembi et al. found that NovaBone putty outperforms the hydroxyapatite bone graft Frios Algipore (Friadent, Germany) in treating intra‐bony defects.^[^ ^163^ ^]^ In contrast, Koduru et al. reported that the nanohydroxyapatite bone graft Sybograf (Eucare Pharmaceuticals, India) was slightly superior to NovaBone Putty.^[^ ^159^ ^]^ Despite the heterogeneity of these clinical trials, they all showed significant improvements when NovaBone Putty was used compared to the baseline. On the whole, NovaBone putty is deemed an established regenerative technology in periodontitis treatment, but further investigations and long‐term studies are necessary to understand its potential benefits and limitations fully.

#### 2.4.2 C‐Blast Putty

C‐Blast Putty (Citagenix Inc, Canada) comprises a demineralized bone matrix in a carboxymethyl cellulose carrier.^[^ ^253^ ^]^ DBM is an allograft sourced from approved tissue banks with osteoconductive and osteoinductive properties.^[^ ^253^ ^]^ To prepare the DBM, an acidic solution is used to remove the mineral components while retaining collagen, non‐collagen proteins, osteoinductive growth factors (such as bone morphogenetic proteins), and 1%−6% residual calcium phosphate mineral, along with traces of cell debris.^[^ ^250^ ^]^ Additionally, the acidic treatment eliminates the original cells and eventual bacteria in the allogenic bone, reducing the risk of an immune response.^[^ ^250^ ^]^ The carboxymethyl cellulose carrier is added to achieve putty consistency, enabling easy molding and optimal adaptation of the material to the defect site while maintaining its compactness.^[^ ^250^ ^]^ A randomized clinical trial conducted by Temraz and colleagues investigated the effectiveness of combining OFD with either C‐Blast Putty or an amnion chorionic membrane (ACM) for treating intra‐bony defects.^[^ ^253^ ^]^ At 6 months, the clinical and radiographic outcomes improved significantly for both groups, including bone defect area change (ACM: 5.17 ± 3.29 mm^2^; C‐Blast Putty: 4.45 ± 3.57 mm^2^), clinical attachment level gain (ACM: 2.25 ± 0.75 mm; C‐Blast Putty: 2.73 ± 0.85 mm) and probing pocket depth reduction (ACM: 3.18 ± 0.85 mm; C‐Blast Putty: 3.45 ± 1.08 mm).^[^ ^253^ ^]^ No statistical differences were observed between the groups in any of these parameters, indicating the comparable clinical efficacy of these materials.^[^ ^253^ ^]^ However, most of the defects in the studies included 2‐wall and 3‐wall intra‐bony defects: their morphology can help to prevent membrane collapse, thus ensuring stable spacing and promoting bone regeneration. Therefore, the putty still appears to be an attractive option, especially if the destruction of periodontal tissues is more extensive. Nonetheless, further clinical data on C‐Blast Putty are needed to evaluate its efficacy in periodontal regeneration properly.

#### 2.4.3 MaxResorb Inject

**Figure 5:**
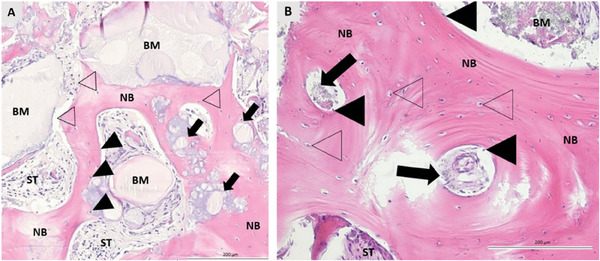
Bone biopsy at six months after augmentation. BM = biomaterial; NB = newly formed bone; ST = soft tissue; No filling triangles = osteocytes; Black filling triangles = osteoblasts; black filling arrows = residual particles of BM. A) MaxResorb Inject. The BM is in direct contact with NB and ST. NB formation begins at the boundary between the BM and the defect. Osteoblasts were detected at the boundary between BM and NB. The remaining granules are integrated into the NB. B) Cerabone. The NB is rich in osteocytes. Osteoblasts were detected at the interface between BM and NB. NB surrounds residual particles of BM. The ST mainly contains fibroblast. Reproduced under terms of the CC‐BY 4.0 license.^[^ ^145^ ^]^ Copyright 2022, The Authors, published by MDPI.

MaxResorb Inject (Botiss Biomaterials, Germany) is an injectable paste obtained from a water‐based gel containing nano‐hydroxyapatite particles and biphasic granules.^[^ ^138^, ^145^ ^]^ The granules contain hydroxyapatite and β‐tricalcium phosphate in a 60:40 ratio, accounting for 16.5% of the paste.^[^ ^138^, ^145^ ^]^ β‐TCP is quickly resorbed, allowing for its substitution and formation of new bone by releasing calcium and phosphate ions, while HA resorbs slowly and provides graft stability.^[^ ^138^, ^254^ ^]^ In a randomized clinical trial, MaxResorb Inject was tested against a bovine xenograft (Cerabone, Botiss Biomaterials, Germany) in alveolar ridge preservation after tooth extraction.^[^ ^145^ ^]^ The histomorphometric analysis showed that, in both groups, bone growth started at the boundaries between the biomaterial and the pristine bone and there was no inflammatory reaction after 6 months (**Figure** **5**).^[^ ^145^ ^]^ There were similar results for new bone formation (MaxResorb Inject: 26.47 ± 14.71%, Cerabone: 30.47 ± 16.39%) and residual biomaterial (MaxResorb Inject: 13.1 ± 14.07%, Cerabone: 17.89 ± 11.81%).^[^ ^145^ ^]^ Moreover, statistically significant improvements were found for the soft tissue percentage (MaxResorb Inject: 60.43 ± 12.73%, Cerabone: 51.64 ± 14.63%).^[^ ^145^ ^]^

In a similar trial, Tomas et al. found that MaxResorb Inject (Test group) and the bovine xenograft Bio‐Oss (Geistlich, Germany – Control group) yield comparable results, not only for newly formed bone (Test: 39.91 ± 8.49%, Control: 41.73 ± 13.99%) and residual biomaterial (Test: 28.61% ± 11.38%, Control: 31.72% ± 15.52%) but also for the soft tissue percentage (Test: 31.49% ± 11.09%, Control: 26.54% ± 7.25%).^[^ ^138^ ^]^ These results indicate that MaxResorb Inject is a valid alternative to xenografts like Bio‐Oss and Cerabone, which are widely employed in periodontology with performances close to autogenous bone.

#### 2.4.4 DBX Putty

Demineralized bone matrix – DBX Putty (MTF Biologics, USA) comprises granulated allogenic cortical bone (31% by weight) in a 4% sodium hyaluronate carrier with a pH of 7.2.^[^ ^255^, ^256^ ^]^ Sodium hyaluronate plays a pivotal role in cellular proliferation, migration, and adhesion and angiogenesis.^[^ ^257^, ^258^ ^]^ In vitro studies have proved the enhancement of osteogenic differentiation and alkaline phosphatase activity with DBX Putty application.^[^ ^259^ ^]^ However, in vitro and animal models highlighted that while DBX Putty is osteoconductive, it shows variable osteoinductivity.^[^ ^260^, ^261^ ^]^ This can be ascribed to the donors’ variability, translating into less predictable clinical outcomes.^[^ ^260^ ^]^ Nonetheless, a randomized clinical trial on periodontal intraosseous defects showed that DBX Putty can improve both hard and soft tissue parameters and compared the results with those obtained with DFDBA (Control group) and DBX paste (26% bone content paste in 2% sodium hyaluronate – MTF Biologics, USA).^[^ ^255^ ^]^ After six months, probing pocket depth reduction (DFDBA: 2.8 ± 1.8 mm, DBX Paste: 3.6 ± 1.5 mm, DBX Putty: 2.3 ± 1.3 mm), clinical attachment level gain (DFDBA: 2.4 ± 1.8 mm, DBX paste: 2.9 ± 1.9 mm, DBX Putty: 1.6 ± 1.1 mm) and bone fill (DFDBA: 2.2 ± 1.8 mm, DBX paste: 2.0 ± 1.6 mm, and DBX putty: 2.4 ± 1.0 mm) improved significantly with respect to baseline, even though there were no significant differences among the groups.^[^ ^255^ ^]^ Due to the lack of histological data, it is impossible to draw a definite conclusion about the regenerative capabilities of DBX Putty and DBX Paste, even though it is plausible that they would have histological outcomes similar to the DFDBA.

#### 2.4.5 Ostim

Ostim (Heraeus Kulzer, Germany) is a nanocrystalline hydroxyapatite paste including 35% nanoscopic apatite particles in an aqueous dispersion.^[^ ^243^, ^262^ ^]^ An in vitro study on human periodontal ligament cells demonstrated that Ostim stimulates cell proliferation, with the mitogenic effect being activated by the epidermal growth factor receptor (EGRF) and its downstream targets ERK1/2 and Akt.^[^ ^263^ ^]^ Although significant, the proliferation with Ostim was lower when compared with Emdogain.^[^ ^263^ ^]^ This is due to the different molecular features of Ostim and Emdogain, which mediate cell proliferation and adhesion through different routes.^[^ ^264^ ^]^ An experimental study in humans showed that Ostim increases the synthesis of bone morphogenetic proteins (BMP‐4 and BMP‐7), alkaline phosphatase, and osteocalcin, thus improving bone regeneration.^[^ ^262^ ^]^ Moreover, Ostim enhances angiogenesis and epithelialization by increasing the vascular endothelial growth factor (VEGF).^[^ ^262^ ^]^ In a randomized split‐mouth clinical trial, 2‐wall intra‐bony defects were treated with papilla preservation flap surgery alone (Control group) or in combination with Ostim (Test group).^[^ ^265^ ^]^ After six months, there were statistically significant higher probing pocket depth reduction (Test: 4.3 ± 1.6 mm; Control: 2.9 ± 1.1 mm) and probing bone level gain (Test: 4.3 ± 1.4 mm; Control: 2.6 ± 1.4 mm) in the group treated with Ostim.^[^ ^265^ ^]^ In a similar study, Ostim combined with OFD was tested against OFD alone for treating intra‐bony defects.^[^ ^266^ ^]^ At six months, there was a significant improvement in probing pocket depth reduction (Test: 3.9 ± 1.2 mm; Control: 2.6 ± 1.3 mm) and clinical attachment level gain (Test: 3.6 ± 1.6 mm; Control: 1.8 ± 1.2 mm) in the Ostim group.^[^ ^266^ ^]^ In another randomized clinical trial using a parallel group design, Ostim was compared to Emdogain for treating intra‐bony defects at 6 and 12 months.^[^ ^243^ ^]^ At 12 months, both treatments provided significant improvements in bone levels (Ostim: 1.6 ± 1.2 mm; Emdogain: 1.6 ± 1.3 mm) and probing pocket depth reduction (Ostim: 2.6 ± 1.8 mm; Emdogain: 3.2 ± 1.8 mm) compared to baseline, with no statistically significant differences between the two groups.^[^ ^243^ ^]^ However, the extent to which the enhancement of clinical parameters resulting from applying the nanocrystalline hydroxyapatite paste indicates regeneration of periodontal tissues remains uncertain.^[^ ^267^ ^]^

#### 2.4.6 MinerOss Putty

MinerOss Putty (Biohorizons Implant Systems Inc., USA) consists of a blend of freeze‐dried allogenic mineralized cortical and cancellous chips along with demineralized cortical fibers.^[^ ^268^, ^269^ ^]^ These components are in a 50:50 ratio and encapsulated within a natural allograft collagen carrier.^[^ ^269^ ^]^ A randomized clinical trial aimed to evaluate the efficacy of a combination of mineralized and demineralized allograft compared to a mineralized allograft for alveolar ridge preservation.^[^ ^270^ ^]^ The results showed that the group treated with a combination of mineralized and demineralized allograft (Test group) exhibited a significantly higher rate of new bone formation (36.16% ± 11.91%) compared to the group treated with solely mineralized freeze‐dried bone allograft (Control group), which had a lower rate of new bone formation (24.69% ± 15.92%).^[^ ^270^ ^]^ Additionally, the Test group demonstrated a significantly lower percentage of residual graft material (18.24% ± 12.47%) than the Control group (27.04% ± 13.26%).^[^ ^270^ ^]^ Although MinerOss graft demonstrates osteoconductive properties, previous studies have indicated limited stability and space‐maintaining capabilities.^[^ ^268^ ^]^ Consequently, combining it with a carrier to achieve a putty‐like consistency could offer advantages for its clinical application, but no clinical data are currently available for MinerOss Putty in periodontal regeneration.

#### 2.4.7 RegenerOss Allograft Putty Plus

RegenerOss Allograft Putty Plus (ZimVie, USA) combines cortical demineralized bone matrix (28%) and cancellous mineralized bone chips (20%) within a lecithin carrier.^[^ ^271^ ^]^ The demineralized bone matrix (DBM) used in the product is manufactured and supplied by LifeLink Tissue Bank, which holds certification from the American Association of Tissue Banks.^[^ ^271^ ^]^ DBM is a biomaterial well‐known for its osteoconductive and osteoinductive properties.^[^ ^250^ ^]^ The putty consistency of RegenerOss Allograft Putty Plus enhances its handling characteristics, and the inclusion of the carrier has the potential to improve its osteoinductivity further.^[^ ^272^ ^]^ At present, ZimVie has chosen not to publish clinical studies associated with RegenerOss Allograft Putty Plus, so the information remains limited.

## 3 Discussion

With the growing demand for effective treatment options in periodontitis, regenerative products have emerged as promising tools, showcasing their potential to promote periodontal regeneration. An ideal regenerative product for periodontal regeneration should, in principle, possess volume‐filling properties, restrict epithelial cells, and possibly hinder new biofilm formation.^[^ ^2^, ^45^, ^172^ ^]^ This would help prevent periodontitis progression and achieve regeneration of all periodontal tissues.

Among the regenerative products, Emdogain is commonly used due to extensive supporting clinical evidence.^[^ ^85^, ^86^, ^156^, ^194^, ^195^ ^]^ Despite this, some clinical trials have highlighted how Emdogain does not further improve clinical and radiographic outcomes.^[^ ^123^, ^137^, ^142^, ^143^, ^148^, ^155^, ^196^, ^197^ ^]^ Additionally, Emdogain does not meet all the requirements of an ideal periodontal regenerative material, as it might degrade fast and lead to flap collapse, thus hindering bone regeneration.^[^ ^86^, ^198^ ^]^ Moreover, its adsorption on root surfaces might be negatively influenced by the surrounding blood, which has to be minimized during surgical procedures.^[^ ^223^ ^]^

A potential alternative to Emdogain is the REGROTH dental kit, which has shown superior efficacy in clinical trials conducted by Kitamura.^[^ ^206^ ^]^ However, the same research group carried out all the clinical trials on REGROTH dental kit.^[^ ^201^, ^205^, ^206^ ^]^ Therefore, further studies involving multiple centres and long‐term observations are necessary. Moreover, REGROTH dental kit is a medicinal product that has only been approved by the Japanese Health Authority, i.e., the Pharmaceuticals and Medical Devices Agency, and it is not available worldwide.^[^ ^273^ ^]^ In comparison, Emdogain is a medical device associated with a significantly lower development cost.^[^ ^274^, ^275^ ^]^ Hence Emdogain might still provide greater health value (health benefit divided by product cost) than REGROTH dental kit.

Hyaluronic acid‐based products are now emerging as a clinically relevant treatment alternative due to the material's intrinsic regenerative potential.^[^ ^210^ ^]^ However, the clinical trial by Pilloni et al. demonstrated that the performance of hyaDENT BG falls short of that of Emdogain.^[^ ^222^ ^]^ Furthermore, there is currently no study comparing the efficacy of Gengigel to Emdogain. The clinical literature on hyaluronan hydrogels, particularly Ossigel and Aminogam, remains limited, making it difficult to evaluate their potential for periodontal regeneration thoroughly.

Platelet concentrates have gained popularity in periodontal regeneration due to their autologous nature and high growth factor content.^[^ ^59^ ^]^ However, being autologous, PCs' composition and biological properties may vary from patient to patient, leading to less predictable clinical outcomes. PRP has been used more rarely in recent years due to its expensive production procedure and biochemical modification.^[^ ^71^ ^]^ On the other hand, PRF's use has been extensively documented in the literature. A clinical trial by Gupta showed that both PRF and Emdogain are effective in treating 3‐wall intra‐bony defects, but Emdogain yielded significantly better results in terms of defect resolution (Emdogain: 43.07% ± 12.21; PRF: 32.41% ± 14.61) after six months.^[^ ^85^ ^]^ Nonetheless, complete defect closure was not achieved.^[^ ^85^ ^]^ It can be theorized that PRF combined with other treatments can provide a synergetic effect, however, it appears that combining PRF with Emdogain does not change the clinical outcomes compared to using Emdogain alone.^[^ ^86^ ^]^ Since neither PRF nor Emdogain are rigid, they cannot ensure space maintenance: this might explain why their combination did not improve the outcomes further. Lastly, CGF, I‐PRF, A‐PRF, and T‐PRF have been proposed as new alternatives to PRF due to enhanced growth factor release.^[^ ^75^ ^]^ Nevertheless, clinical studies are currently scarce and not conclusive.

Besides hydrogels and platelet concentrates, bone grafts also play a pivotal role in periodontal regeneration as they can guarantee better space‐maintaining properties, which translates into improved hard tissue metrics. Among these, the xenograft Bio‐Oss is considered a leading product in the field, alongside Emdogain, and has been widely used in the clinics.^[^ ^276^ ^]^ Xenografts stand out for their affordability, availability and predictable clinical results, although they can show variable resorption rate and immunogenicity.^[^ ^119^, ^120^, ^121^ ^]^ However, the most suitable graft type depends on several factors, such as resorption rates, tissue integration, and specific clinical case requirements. For example, autografts offer excellent biocompatibility, but there can be issues with patient morbidity and material availability.^[^ ^118^, ^119^, ^120^, ^121^ ^]^ Allografts provide an alternative even though they may raise concerns related to immune compatibility.^[^ ^118^, ^119^, ^120^, ^121^ ^]^ On the other hand, synthetic bone grafts appear interesting as their physiochemical properties can be tuned.^[^ ^121^ ^]^ Therefore, it is crucial to carefully assess the patient's needs.

Another possibility is that of bone putties as they possess space‐maintaining properties typical of bone grafts, which hydrogels generally lack, while being moldable and more easily adaptable to the defect site.^[^ ^154^, ^250^ ^]^ Among the well‐documented bone putties, there are NovaBone Putty, which is considered an established regenerative product, Ostim and MaxResorb Inject. Notably, Ostim has shown non‐inferiority to Emdogain, demonstrating its potential as a comparable alternative, even though histological evidence is missing.^[^ ^243^, ^267^ ^]^ In addition, MaxResorb Inject has yielded promising results comparable to those of Bio‐Oss.^[^ ^138^ ^]^ Comparatively, information on the clinical performance of C‐Blast Putty, DBX Putty, MinerOss Putty, and RegenerOss Allograft Putty Plus remains scarce, limiting the discussion of these solutions.

Overall, although applying the current regenerative options provides relevant clinical and radiographic results, it should be noted that complete defect closure is hardly achieved, even for the indicated class II furcation and intra‐bony defects.^[^ ^46^ ^]^

Lastly, selecting the clinical study design is equally important as it ensures the production of robust, replicable results that guide evidence‐based decisions. Additionally, clinical trials are the most expensive and time‐consuming phase during the technical transfer of a new product from concept to market.^[^ ^274^, ^275^ ^]^ The randomized clinical trials found in periodontal regeneration use either a split‐mouth design or parallel groups (Table S1, Supporting Information). While the split‐mouth design has the advantage of a smaller sample size and the absence of inter‐subject variability, it is unsuitable for all interventions because potential carryover effects can lead to bias.^[^ ^277^, ^278^, ^279^ ^]^ It is noteworthy that parallel groups can be identified based on the number of patients or defects. The latter can be adopted when some of the patients in the study exhibit more than one defect. Two possible scenarios arise in such cases: multiple sites in the same patient undergoing the same treatment or different ones.^[^ ^67^, ^70^, ^85^, ^93^, ^101^, ^113^, ^116^, ^124^, ^129^, ^132^, ^139^, ^154^ ^]^ Some clinical trials do not specify further how this randomization is performed.^[^ ^81^, ^94^, ^147^, ^233^ ^]^ This approach might introduce further complexities when interpreting the results, and it has been found that most trials in periodontology could benefit from better methodological and reporting quality.^[^ ^278^, ^279^ ^]^ Furthermore, the studies should be designed to be long‐term, with the endpoint measurements being ideally taken at least 1 year postoperatively.^[^ ^280^ ^]^ This was only found in 39.3% of the included trials (Table S1, Supporting Information). The most reliable technique to assess the regeneration capability of a product is histology.^[^ ^280^, ^281^ ^]^ However, animal models are generally used instead due to the associated morbidity.^[^ ^280^, ^281^ ^]^ This can raise problems of interpretation when translating the results to humans. Therefore, other variables must be used, typically direct bone measurements, clinical attachment level gain, and probing pocket depth reduction.

### 3.1 Future of Periodontal Regeneration

Biofilm removal is crucial in the early and advanced stages of periodontitis to ensure good periodontal regeneration and prevent its reoccurrence.^[^ ^282^ ^]^ In recent years, researchers found that medications, and especially host modulators, show great potential in resolving or inhibiting the periodontal inflammatory process, particularly in the early phases of periodontitis combined with non‐surgical periodontal treatment.^[^ ^283^, ^284^, ^285^, ^286^, ^287^, ^288^, ^289^ ^]^ These host modulators include statins like atorvastatin, rosuvastatin, and simvastatin, as well as alendronate and metformin.^[^ ^80^, ^89^, ^92^, ^95^, ^96^, ^98^, ^99^, ^105^, ^290^, ^291^, ^292^, ^293^, ^294^, ^295^, ^296^ ^]^ Statins exhibit anti‐inflammatory and immunomodulatory properties while inhibiting osteoclasts and stimulating bone morphogenetic proteins.^[^ ^297^ ^]^ Metformin, commonly prescribed for type 2 diabetes patients, reduces the inflammatory response and oxidative stress and stimulates osteoblastic activity.^[^ ^298^ ^]^ Alendronate, a bisphosphonate often used to treat osteoporosis, effectively suppresses bone resorption and possesses anti‐inflammatory properties.^[^ ^299^ ^]^ In clinical trials, they have been administered as hydrogels, with a concentration of 1.2% for statins and 1% for alendronate and metformin, demonstrating improvements in clinical and radiographic parameters.^[^ ^283^ ^]^ However, these gels are currently not available on the market.

Regarding the imperative for space maintenance, this becomes vital for the proliferation of bone‐forming cells.^[^ ^172^ ^]^ Indeed, mesenchymal cells have a slower migration rate than epithelial and connective cells.^[^ ^45^ ^]^ Hence, the latter have to be restricted to ensure functional regeneration of all periodontal tissues.^[^ ^276^ ^]^ When a regenerative product cannot guarantee stable spacing in clinical applications, volume‐filling is typically achieved by combining different products.^[^ ^249^ ^]^ Another strategy involves pairing the regenerative materials with membranes to favor mesenchymal cell growth over epithelial cells, according to the principles of guided tissue regeneration.^[^ ^300^, ^301^ ^]^ A variety of membranes are employed in clinical practice, encompassing both non‐resorbable options like Gore‐Tex (W.L. Gore *&* Associates, USA) and resorbable ones, such as the collagen‐based Bio‐Gide (Geistlich, Switzerland) and BioMend Extend (ZimVie, USA).^[^ ^276^ ^]^ Their analysis was, however beyond the scope of this review.

The majority of the biomaterials for periodontal regeneration only partially mimic the composition of periodontal tissues, particularly periodontal ligament fibers.^[^ ^302^, ^303^ ^]^ To achieve functional regeneration, novel bio‐inspired biomaterials replicating the hierarchical structures of periodontal tissues at the micro and nanoscale levels are necessary.^[^ ^302^ ^]^ Emerging technologies include bioactive nanomaterials, multilayered scaffolds, stem cells, bone anabolic agents, and genetic therapies.^[^ ^276^, ^302^, ^304^, ^305^, ^306^ ^]^ Nevertheless, these technologies are still complex and in their early stage. Indeed, further evidence is required to fulfil the regulatory requirements for implementation in a clinical context.

Eventually, it is essential to have a proper clinical study design for evaluating the efficacy of the treatment approaches. First, the quality of the clinical trials can be improved by adhering strictly to the Consolidated Standards of Reporting Trials (CONSORT) 2010 statement, beyond the Declaration of Helsinki.^[^ ^307^, ^308^ ^]^ Then, it is crucial to define a primary outcome beforehand, as the study should be built accordingly to avoid wasting resources.^[^ ^279^ ^]^ Lastly, attention should be paid to the fact that periodontal regeneration responds differently to different kinds of defects, with class II furcation defects, 2‐wall and 3‐wall defects yielding the best and most predictable results.^[^ ^43^, ^281^ ^]^ The clinical trials usually differentiate between furcation and intra‐bony defects, but further classification of these is not always ensured. However, while designing separate and specific clinical trials can provide more reliable results, this also leads to higher costs and limits the generalizability of the outcomes to a broader population. Therefore, it is necessary to carefully consider the research objectives, available resources, and clinical relevance of the findings.

## 4 Conclusion

Periodontitis is a widespread dental disease that has a significant impact worldwide. Its high incidence emphasizes the urgent need for effective treatments. In this regard, regenerative products have emerged as promising tools. Among these, Emdogain (Straumann AG, Switzerland) is a common practice, with extensive clinical evidence supporting its use for periodontal regeneration, alone or in combination with the xenograft Bio‐Oss (Geistlich, Switzerland). However, alternative products such as platelet concentrates, REGROTH dental kit (Kaken Pharmaceutical Co., Ltd, Japan), hyaluronan‐based hydrogels, and putties have shown great potential but require further studies and clinical data to establish their effectiveness and superiority concerning the conventional regenerative options. Host modulator hydrogels are also considered remarkable, particularly in the early phases of periodontitis, although they are still under investigation and, as such, not yet available on the market

Overall, despite the positive results that it is possible to achieve, complete defect closure is often not obtained, even for indicated defects such as class II furcation defects and intra‐bony defects, while class III furcation defects remain challenging to regenerate. To date, none of the available regenerative biomaterials for periodontal regeneration possess all the ideal characteristics, and research is oriented towards novel bio‐inspired biomaterials replicating the hierarchical structures of periodontal tissues. Besides the biomaterials perspective, our review suggests that there is room for improvement in the methodological and reporting quality of the clinical trials, which should also optimize time and costs. To do so, following the CONSORT 2010 statement is highly advisable.

In conclusion, while the current regenerative products are valuable options for periodontal regeneration, ongoing research and development are needed to refine their efficacy, expand their availability, and overcome the existing limitations in the field to achieve optimal clinical and radiographic outcomes.

## 5 Experimental Section

MEDLINE was searched through the PubMed interface to identify the regenerative technologies clinically adopted for periodontal regeneration. The Boolean operator “AND” was used to merge keywords, resulting in the formation of the following search string: (Periodontal[Title/Abstract]) AND (Regeneration[Title/Abstract]). The search was restricted to clinical trials, the English language, and the year of publication, 2011–2023. The additional regenerative products were searched on the leading dental companies’ websites. Once the products were identified, their brand name was searched on MEDLINE to get a comprehensive overview of the available literature. The full text of the clinical trials was assessed, and the exclusion criteria were: 1) Absence of testing on humans; 2) Absence of regenerative products; 3) Treatments including membranes only; 4) Article not available; 5) Insufficient information, e.g., number of patients, treatment method, quantitative results, incomplete results (i.e., mean value without standard deviation); 6) Number of patients < 8.

**Figure 6:**
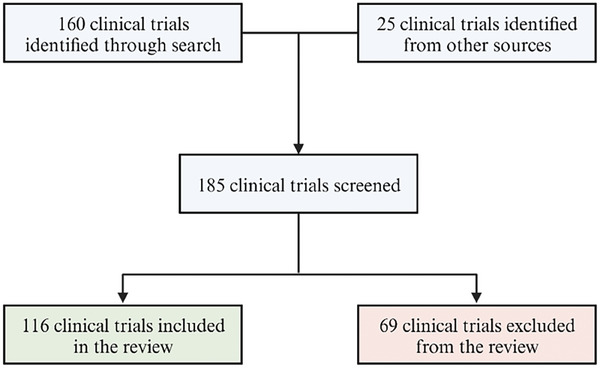
Graphical representation of clinical trials found on MEDLINE with the search terms (Periodontal[Title/Abstract]) AND (Regeneration[Title/Abstract]) for the period 2011–2023.

A flowchart describing the selection process is reported in **Figure** **6**.

## Conflict of Interest

The authors declare no conflict of interest.

## Supporting information

Supporting Information
