# Supplementary material for: From Basic Science to Clinical Practice: A Review of Current Periodontal/Mucogingival Regenerative Biomaterials
Source: Adv Sci (Weinh). 2024 Feb 21;11(17):2308848. doi: 10.1002/advs.202308848 (PMC11077667; doi:10.1002/advs.202308848)
Supplement: Supplementary file 1 — Supporting Information [file ADVS-11-2308848-s001.pdf]

ADVANCED SCIENCE
Open Access

Supporting Information

for *Adv. Sci.*, DOI 10.1002/advs.202308848

From Basic Science to Clinical Practice: A Review of Current Periodontal/Mucogingival Regenerative Biomaterials

*Angela De Lauretis, Øystein Øvrebø, Mario Romandini, Ståle Petter Lyngstadaas, Filippo Rossi and Håvard Jostein Haugen**

Supporting Information

Table S1. Treatment groups, addressed defect, randomization, duration, number of patients, variables assessed and conclusion of the clinical studies included in the review.

*Angela De Lauretis, Øystein Øvrebø, Mario Romandini, Ståle Petter Lyngstadaas, Filippo Rossi, Håvard Jostein Haugen**

Clinical study	Treatment groups	Defect	Randomization	Duration [months]	Number of patients	Variables assessed	Conclusion
[67]	**1**: PRP<br>**2**: PRP +Bio-Oss®	3-wall and combined 2-3 wall intra-bony defects	Parallel (defect-based)	9	14	GI, PI, PPD, CAL, BDD	Both groups show significant clinical improvements with respect to baseline, but no statistically significant differences between the groups.
[68]	**1**: β-TCP<br>**2**: β-TCP + PRP	Intra-bony defects	Split-mouth	9	20	PPD, CAL, BDD	Clinical and radiographic improvements more significant in group 2.
[69]	**1**: Healiguide® membrane<br>**2**: PRP<br>**3**: PRP + Collacote® sponge	Apicomarginal defects	Parallel	12	30	PPD, CAL, GMP	All groups show significant clinical improvements with respect to baseline, but no statistically significant differences between the groups.
[70]	**1**: OFD + PRF<br>**2**: OFD + PRP<br>**3**: OFD	Mandibular class II furcation defects	Parallel (defect-based)	9	37	PI, BOP, PPD, CAL, HCAL, GMP, BDD	Clinical and radiographic improvements more significant in groups 1 and 2.

[76]	**1**: PRF + Periocol® membrane<br>**2**: Periocol® membrane	3-wall intra-bony defects	Split-mouth	9	16	PPD, CAL, GMP, BDD	Clinical and radiographic improvements more significant in group 1.
[77]	**1**: Autogenous demineralized dentin matrix + PRF + Bio-Gide® membrane<br>**2**: Bio-Oss® + PRF + Bio-Gide® membrane	Post extraction sockets	Parallel	18	40	ISQ, MBR	Both groups show significant clinical improvements with respect to baseline, but no statistically significant differences between the groups.
[78]	**1**: OFD + PRF + Ascorbic Acid<br>**2**: OFD + PRF	2- or 3-wall intra-bony defects	Parallel	6	20	CAL, PPD, GMP, FMBS, BDD, BDe	Both groups show significant clinical improvements with respect to baseline. Group 1 shows more significant improvements in GMP and BDe.
[79]	**1**: NovaBone® Dental Morsels + PRF<br>**2**: PRF	Intra-bony defects	Split-mouth	6	20	PPD, CAL, GMP, BDD	Clinical and radiographic improvements more significant in group 1.
[80]	**1**: PRF<br>**2**: PRF + 1% Alendronate gel	Mandibular class II furcation defects	Split-mouth	6	20	BDV, BDD, BDW, PPD, CAL, HPD	Clinical and radiographic improvements more significant in group 2.
[81]	**1**: NovaBone® Putty<br>**2**: PRF	Mandibular class II furcation defects	Parallel (defect-based)	9	15	GI, PI, PDD, CAL, GMP, HPD	Clinical improvements more significant in group 1.
[82]	**1**: PRF<br>**2**: ABG	3-wall intra-bony defects	Parallel	9	20	GI, PPD, CAL, BDe	Both groups show significant clinical and radiographic improvements. Group 2 shows more significant radiographic outcomes.
[83]	**1**: Tutodent® Chips<br>**2**: PRGF<br>**3**: PRF	2- and 3-wall intra-bony defects	Parallel	9	30	GI, PI, PPD, CAL, BDD, PDGF-BB and VEGF concentrations	Clinical improvements more significant in group 1.

[84]	**1**: L-PRF + Bio-Oss®<br>**2**: Evolution® Membrane + Bio-Oss®	1-, combined 1-2 and 2-wall intra-bony defects	Parallel	12	62	FMPS, FMBS, PPD, CAL, GPM, BDD	Both groups show significant clinical improvements with respect to baseline. Group 2 shows more significant improvements in PDD.
[85]	**1**: Emdogain®<br>**2**: PRF	3-wall intra-bony defects	Parallel (defect-based)	6	30	PPD, CAL, BDD, BDAn	Clinical and radiographic improvements more significant in group 1.
[86]	**1**: Emdogain®<br>**2**: Emdogain® + PRF	Intra-bony defects	Split-mouth	6	14	GI, PI, PPD, CAL, GPM, BDD, BDW, BDAn	Both groups show significant clinical and radiographic improvements with respect to baseline, but no statistically significant differences between the groups.
[87]	**1**: Primary closure<br>**2**: PRF + primary closure	Post extraction sockets	Parallel	24	31	Pain, Swelling, PPD, BDe	Clinical improvements more significant in group 2.
[88]	**1**: OFD + PRF<br>**2**: OFD + OSSIX plus® membrane<br>**3**: OFD	2- and 3-wall intra-bony defects	Split-mouth	12	30	PI, GI, BOP, PPD, CAL, TM, WH, BDD, ACR	Clinical and radiographic improvements more significant in group 1 and 2 than 3, but no significant differences between groups 1 and 2.
[89]	**1**: Access therapy<br>**2**: Access therapy + PRF<br>**3**: Access therapy + PRF + 1% Alendronate gel	Mandibular class II furcation defects	Parallel	9	72	PI, mSBI, PPD, CAL, HCAL, BDD, BDF	Clinical and radiographic improvements more significant in group 3.
[90]	**1**: OraGRAFT® + PRF<br>**2**: OraGRAFT® + saline	2- and 3-wall intra-bony defects	Split-mouth	12	30	PPD, CAL, GMP, BDD, ACR, DR	Clinical and radiographic improvements more significant in group 1.
[91]	**1**: PRF<br>**2**: PRF + Bio-Oss®	2- and 3-wall intra-bony defects	Split-mouth	6	17	PPD, CAL, GMP, BDF, ACR, PI, GSBI	Clinical and radiographic improvements more significant in group 2.
[92]	**1**: OFD + PRF<br>**2**: OFD + PRF +1.2% Atorvastatin	3-wall intra-bony defects	Parallel	9	96	PI, mSBI, PPD, CAL, GMP, BDD	Clinical improvements more significant in groups 1 and 2 than 3, but no significant

	gel<br>**3**: OFD						differences berween groups 1 and 2. Radiographic improvements more significant in group 2.
[93]	**1**: OFD + PRF<br>**2**: OFD + PRF + SyboGraf™<br>**3**: OFD	3-wall intra-bony defects	Parallel (defect-based)	9	62	PPD, CAL,GMP, BDD, BDF, mSBI, PI	Clinical and radiographic improvements more significant in group 1 and 2 than 3.
[94]	**1**: OFD<br>**2**: OFD + PRF<br>**3**: OFD + PRF + DFDBA	Mandibular class II furcation defects	Parallel (defect-based)	9	46	PPD, CAL, GMP, HPD, BDF, HBDF, BDW	Clinical and radiographic improvements more significant in groups 2 and 3 than 1. Group 3 shows more significant improvements in BDF than group 2.
[95]	**1**: OFD<br>**2**: OFD + PRF<br>**3**: OFD + PRF + 1.2% Rosuvastatin gel	2- and 3-wall intra-bony defects	Parallel	9	90	PI, mSBI, PPD, CAL, BDD	Clinical and radiographic outcomes more significant in groups 2 and 3 than 1.
[96]	**1**: OFD<br>**2**: OFD + PRF<br>**3**: OFD + 1% Metformin gel<br>**4**: OFD + PRF + 1% Metformin gel	3-wall intra-bony defects	Parallel	9	120	PI, mSBI, PPD, CAL, GMP, BDD	Clinical and radiographic outcomes more significant in group 4 than the other groups. Groups 2 and 3 show more significant clinical and radiographic improvements than group 1.
[97]	**1**: Primary closure<br>**2**: L-PRF + primary closure	Post extraction sockets	Split-mouth	3	17	Pain, BDe, WH	Group 2 shows more significant BDe only.
[98]	**1**: OFD + Placebo gel<br>**2**: OFD + PRF + SyboGraf™<br>**3**: OFD + PRF + Sybograf™ + 1.2% Rosuvastatin gel	Mandibular class II furcation defects	Parallel	9	105	mSBI, PI, PPD, CAL, HCAL, BDD, BDF	Clinical and radiographic improvements more significant in group 3.

[99]	**1**: Access therapy<br>**2**: Access therapy + PRF<br>**3**: Access therapy + PRF + 1% Alendronate gel	3-wall intra-bony defects	Parallel	9	90	PI, mSBI, PPD, CAL, GMP, BDD	Clinical and radiographic improvements more significant in group 3.
[100]	**1**: SRP + PRF<br>**2**: SRP	Deep periodontal pockets	Split-mouth	6	12	PI, GI, BOP, PPD, CAL, GMP, biochemical parameters (Col-1, TGF-β)	Clinical improvements more significant in group 1.
[101]	**1**: OFD + PRF<br>**2**: OFD	3-wall intra-bony defects	Parallel (defect-based)	9	35	PI, mSBI, PPD, CAL, GMP, BDD	Clinical and radiographic improvements more significant in group 1.
[102]	**1**: PRF + Bio-Oss® + Collagen membrane;<br>**2**: Bio-Oss® + Collagen Membrane	Intra-bony defects	Split-mouth	24	14	PPD, CAL, BDD, BDW	Clinical improvements more significant in group 1.
[103]	**1**: PRF + OFD<br>**2**: OFD	2- and 3-wall intra-bony defects	Parallel	9	22	PPD, CAL, GMP, FMBS, FMPS, BDD, BDF	Clinical improvements more significant in group 1.
[104]	**1**: OFD + ABG + L-PRF<br>**2**: OFD + ABG<br>**3**: OFD	Mandibular class II furcation defects	Parallel	6	54	PPD, HCAL, CAL, GMP, BDD	Clinical improvements more significant in group 1.
[105]	**1**: PRF<br>**2**: PRF + 1% Metformin gel	Mandibular and maxillary class II furcation defects	Split-mouth	12	21	CAL, HPD, PPD, BDV	Clinical and radiographic improvements more significant in group 2.
[106]	**1**: NovaBone® Putty<br>**2**: NovaBone® Putty + PRF	Intra-bony defects	Parallel	6	20	PI, GI, PPD, CAL, BDD	Both groups have significant clinical and radiographic improvements with respect to baseline. Group 2 shows significantly better CAL and PPD.
[107]	**1**: NovaBone® Putty<br>**2**: NovaBone® Putty + PRF	2- and 3-wall intra-bony defects	Split-mouth	9	10	PI, GI, PPD, CAL, BDF	Both groups show significant clinical and radiographic improvements with respect

							to baseline. Group 2 shows significantly better BDF.
[112]	**1**: CGF + modified Ward's incision<br>**2**: Modified Ward's incision	Post extraction sockets	Split-mouth	3	30	BDD, BDW, Bde	Outcomes improvements are more significant in group 1.
[113]	**1**: Flap surgery<br>**2**: Flap surgery + CGF<br>**3**: Flap surgery + Bio-Oss®<br>**4**: Flap surgery + CGF + Bio-Oss®	1-wall intra-bony defects	Parallel (defect-based)	12	54	PPD, CAL	Clinical improvements more significant in groups 2, 3 and 4 than 1. Groups 3 and 4 show more significant clinical outcomes than group 2, but no statistically significant differences between them.
[114]	**1**: DFDBA + CGF<br>**2**: CGF	Intra-bony defects	Split-mouth	6	10	PI, modified GI, PPD, CAL, BDA	Both groups show significant clinical and radiographic improvements with respect to baseline, but no statistically significant differences between the groups.
[115]	**1**: A-PRF<br>**2**: No intervention	Post extraction sockets	Split-mouth	3	10	PPD, CAL, GMP, Pain, Swelling, WH	Pain and swelling are significantly reduced in group 1. There is no significant difference in clinical outcomes between the groups.
[116]	**1**: OFD<br>**2**: OFD + PRF<br>**3**: OFD + T-PRF	3-wall intra-bony defects	Parallel (defect-based)	9	38	PI, PPD, CAL, BDF	Clinical improvements more significant in groups 2 and 3 than 1, but no statistically significant difference between groups 2 and 3.
[117]	**1**: I-PRF + DFDBA<br>**2**: DFDBA	2- and 3-wall intra-bony defects	Parallel	9	20	CAL, PPD, GMP, FMPS, FMBS, BDD, BDF	Clinical and radiographic improvements with respect to baseline, but no statistically significant differences between the groups.
[123]	**1**: L-PRF + ABG<br>**2**: PrefGel® + Emdogain® + ABG	1-, combined 1-2 and 2-wall intra-bony defects	Parallel	12	44	PPD, CAL, GMP, BDD	Clinical and radiographic improvements with respect to baseline, but no

							statistically significant differences between the groups.
[124]	**1**: SRP + ABG<br>**2**: SRP	Post extraction sockets	Parallel (defect-based)	12	51	Pain, Swelling, Complication %, BDD, PPD, CAL	Clinical and radiographic improvements more significant in group 1.
[129]	**1**: REGROTH® Dental Kit + Bio-Oss®<br>**2**: REGROTH® Dental Kit	1-, 2- and 3-wall intra-bony defects	Parallel (defect-based)	6	32	CAL, PPD, GMP, BOP, TM, BDF	Clinical improvements more significant with respect to baseline in both groups, but group 1 yields more significant radiographic outcomes.
[130]	**1**: Regeneration (Combinations of PrefGel®, Emdogain®, GoreTex®/Guidor®/Bio-Gide® + Bio-Oss®)<br>**2**: Tooth extraction and replacement	Deep intra-bony defects	Parallel	120	50	CAL, PPD, tooth prognosis, patient reported outcomes	The complexity of the treatment limits widespread application to the most complex cases. Still, regeneration (group 1) could change the prognosis of hopeless teeth.
[131]	**1**: Regeneration (Combinations of PrefGel®, Emdogain®, GoreTex®/Guidor®/Bio-Gide® + Bio-Oss®)<br>**2**: Tooth extraction and replacement	Deep intra-bony defects	Parallel	60	50	FMPS, FMBS, PPD, CAL, TM, BDD	The complexity of the treatment limits widespread application to the most complex cases. Still, regeneration (group 1) could change the prognosis of hopeless teeth.
[132]	**1**: PDLSCs sheets + Bio-Oss®<br>**2**: Bio-Oss®	2- and 3-wall intra-bony defects	Parallel (defect-based)	12	30	CAL, BDD, PPD, GMP	Both groups show significant clinical and radiographic improvements with respect to baseline, but no statistically significant differences between the groups.
[133]	**1**: Bio-Oss® + modified perforated Bio-Gide® membrane<br>**2**: Bio-Oss® + Bio-Gide® membrane	1-, 2- and 3-wall intra-bony defects	Split-mouth	12	15	PPD, CAL, GMP, BDD, ACR, BDF	Both groups show significant clinical and radiographic improvements with respect to baseline. Group 1 shows more significant BDF.

[134]	**1**: Bio-Oss® + modified perforated Bio-Gide® membrane<br>**2**: Bio-Oss® + Bio-Gide® membrane	1-, 2- and 3-wall intra-bony defects	Split-mouth	12	15	Pain, PPD, CAL, GMP, BDD, BDe	Both groups show significant clinical and radiographic improvements with respect to baseline, but no statistically significant differences between the groups.
[135]	**1**: Minimally invasive surgical technique modified in suture<br>**2**: Minimally invasive surgical technique modified in suture + Bio-Oss® + Bio-Gide® membrane	Intra-bony defects	Parallel	12	36	PPD, CAL, GMP, BDD	Both groups show significant clinical and radiographic improvements with respect to baseline, but no statistically significant differences between the groups.
[136]	**1**: Bio-Oss® + Bio-Gide® membrane<br>**2**: THE Graft + Bio-Gide® membrane	Post extraction sockets	Parallel	4	100	BDW, BDD, BDV, STD	Both groups show significant soft and hard tissue improvements with respect to the baseline, but no statistically significant differences between the groups.
[137]	**1**: PrefGel® + Emdogain® + Bio-Oss®<br>**2**: Bio-Oss® + Bio-Gide®	Non-contained intra-bony defects (at least a 70% 1-wall component and a residual 2- to 3-wall component)	Parallel	12	40	FMPS, FMBS, PPD, CAL, GMP	Both groups show significant clinical improvements with respect to baseline, but no statistically significant differences between the groups.
[138]	**1**: MaxResorb Inject®<br>**2**: Bio-Oss®	Post extraction sockets	Parallel	6	38	Histology (newly formed bone, residual biomaterial, soft tissue)	Both groups show significant results with respect to baseline, but no statistically significant differences between the groups.
[139]	**1**: REGROTH® Dental Kit<br>**2**: REGROTH® Dental Kit + Bio-Oss®	1-, 2. and 3-wall intra-bony defects	Parallel (defect-based)	48	32	CAL, PPD, GMP, BOP, TM, BDF	Both groups show significant clinical improvements with respect to baseline. Group 2 shows more significant radiographic outcomes.

[140]	**1**: Bio-Oss® Collagen + PDL-MSCs<br>**2**: Bio-Oss® Collagen	1- and 2- wall intra-bony defects	Parallel	12	20	PPD, CAL, GMP	Both groups show significant clinical improvements with respect to baseline, but no statistically significant differences between the groups.
[141]	**1**: Tutodent® membrane + Tutodent® Chips	Combined 1- and 2-wall intra-bony defects	One group only	36	30	PPD, CAL, histology	Significant clinical improvements with respect to baseline. Histology reveals regeneration of cementum and periodontal ligament, but without bone formation.
[142]	**1**: Non-incised surgical papillae surgery + PrefGel® + Emdogain®<br>**2**: Non-incised surgical papillae surgery + PrefGel® + Emdogain® + Cerabone®	1- and 2-wall intra-bony defects with a supra-alveolar componenet	Parallel	12	24	BOP, CAL, PPD, GMP, TP, KT, SUPRA-AG	Both groups show significant clinical improvements with respect to baseline, but no statistically significant differences between the groups.
[143]	**1**: Entire papilla preservation<br>**2**: Entire papilla preservation + PrefGel® + Emdogain® + Cerabone®	2- and 3-wall intra-bony defects	Parallel	12	30	Pain, PPD, CAL, GMP	Both groups show significant clinical improvements with respect to baseline, but no statistically significant differences between the groups.
[144]	**1**: Cerabone® + Collprotect® membrane + Amoxicillin (twice daily for 7 days)<br>**2**: Cerabone® + Collprotect® membrane	Intra-bony defects	Parallel	12	41	CAL, PPD, GMP, BDD, BDW	Both groups show significant clinical and radiographic improvements with respect to baseline, but no statistically significant differences between the groups.
[145]	**1**: MaxResorb Inject®<br>**2**: Cerabone®	Post extraction sockets	Parallel	6	41	Histology (newly formed bone, residual biomaterial, soft tissue)	Both groups show significant results with respect to baseline. Group 1 shows significantly higher soft tissue percentage.

[146]	**1**: PrefgGel® + ABM/PepGen P-15®<br>**2**: PrefGel® + TRI1/TRI2 GoreTex® membrane	Intra-bony defects	Split-mouth	6	15	PPD, CAL, GMP, BDD, BDF, IL-β1 concentration, IL-β6 concentration	Both groups show significant clinical and radiographic improvements with respect to baseline. Group 1 shows significantly higher bone fill.
[147]	**1**: Bio-Gen® graft + BioCollagen® membrane<br>**2**: Bio-Gen® graft + BioCollagen® membrane + low level laser therapy	Mandibular and maxillary class II furcation defects	Parallel (defect-based)	6	25	PPD, CAL, HPD, OC level, ALP level	Clinical improvements more significant in group 2.
[148]	**1**: THE Graft + Emdogain®<br>**2**: THE Graft	1-wall intra-bony defects	Parallel	24	42	Pain, PPD, CAL, BDD, BDW	Both groups show significant clinical and radiographic improvements with respect to baseline. Group 1 shows a significantly lower severity of pain.
[149]	**1**: PrefGel® + hyaDENT BG + THE Graft	1-, 2- and 3-wall intra-bony defects	One group only	6	23	CAL, PPD, GMP	Significant clinical improvements with respect to baseline.
[150]	**1**: Immediate loading + OraGRAFT® + Collagen membrane<br>**2**: Loading after 3 months + OraGRAFT® + Collagen membrane	Post extraction sockets with implant placement	Parallel	12	60	Implant survival rate and stability, BDD	Both groups show significant radiographic bone level improvements, success rate and stability with respect to baseline.
[151]	**1**: AlloGro® + Omega 3 + aspirin<br>**2**: AlloGro® + placebo	Class II furcation defects	Parallel	6	40	PI, GI, BOP, PPD, CAL, IL-1β and IL-10 concentration	Clinical improvements more significant in group 1.
[152]	**1**: OFD + PerioGlas®<br>**2**: OFD	1-, 2- and 3-wall intra-bony defects	Split-mouth	9	8	PI, GI, PPD, CAL, GMP, BDF, ACR	Both groups show significant clinical and radiographic improvements with respect to baseline. Group 1 shows significantly higher bone fill.

[153]	**1**: PerioGlas® + surgery<br>**2**: Surgery	3-wall intra-bony defects	Split-mouth	12	12	PI, GI, PPD, GMP, CAL, TM, BDD	Clinical and radiographic improvements more significant in group 1.
[154]	**1**: NovaBone® Dental Putty<br>**2**: Perioglas®	Mandibular class II furcation defects	Parallel (defect-based)	6	28	PPD, GMP, CAL, BDD, BDW, HBDD	Both groups show significant clinical and radiographic improvements with respect to baseline. Groups 2 shows significantly higher horizontal defect fill.
[155]	**1**: PrefGel® + Emdogain®<br>**2**: PrefGel® + Emdogain® + Bone Ceramic®	1-, 1- to 2-, and 2-wall intra-bony defects	Parallel	12	52	PPD, CAL, GMP, BDD	Both groups show significant clinical and radiographic improvements with respect to baseline, but no statistically significant differences between the groups.
[156]	**1**: Emdogain®<br>**2**: Bone Ceramic®<br>**3**: Emdogain® + Bone Ceramic®	Mandibular class II furcation defects	Parallel	12	41	PI, GI, GMP, CAL, HCAL, PPD	All groups show significant clinical improvements with respect to baseline, but no statistically significant differences between the groups.
[157]	**1**: OsteoGen®<br>**2**: OsteoGen® + Alendronate solution	2- and 3-wall intra-bony defects	Split-mouth	6	15	PPD, CAL, BDD, BDF	Both groups show significant clinical and radiographic improvements with respect to baseline. Group 2 shows significantly higher bone fill.
[158]	**1**: Gengigel® Prof syringes + SyboGraf™<br>**2**: SyboGraf™	2-, 2- to 3-, and 3-walled intra-bony defects	Split-mouth	12	8	PPD, CAL, BDD, ACR, BDF	Clinical and radiographic improvements more significant in group 1.
[159]	**1**: SyboGraf™<br>**2**: NovaBone® Putty	Intra-bony defects	Parallel	9	20	PI, GI, mSBI, PPD, CAL, GMP, BDD	Both groups show significant clinical and radiographic improvements with respect to baseline. Group 1 appears slightly superior in relation to clinical parameters.
[160]	**1**: PrefGel® + platelet concentrate + Ceros® TCP granules + Resolut	1-, 2-, 3-wall and combined intra-bony defects	Split-mouth	84	25	PBI, BOP, GMP, PPD, CAL	Both groups show significant clinical improvements with respect to baseline.

	XT® membrane<br>**2**: PrefGel® + Ceros® TCP granules + Resolut XT® membrane						Adding platelet concentrate in group 1 may negatively influence the long-term stability.
[161]	**1**: β-TCP + sodium acetate buffer<br>**2**: GEM 21S (0.3mg/mL rhPDGF-BB) + sodium acetate buffer<br>**3**: GEM 21S (1.0 mg/mL rhPDGF-BB) + sodium acetate buffer	2- and 3-wall intra-bony defects	Parallel	36	83	PPD, CAL, BDF, BDD	Clinical and radiographic improvements more significant in group 2.
[162]	**1**: OFD + MD05<br>**2**: OFD	1- and 2- wall intra-bony defects	Parallel	6	20	PPD, CAL, GMP, histology	Both groups show significant clinical improvements with respect to baseline, but no statistically significant differences between the groups. Non-meaningful histological evaluation.
[163]	**1**: Frios® Algipore®<br>**2**: NovaBone® Putty	Intra-bony defects	Split-mouth	6	11	PI, GI, GSBI, PPD, CAL, GMP, BDF	Both groups show significant clinical and radiographic results with respect to baseline. Group 2 shows significantly higher defect fill.
[164]	**1**: Flap surgery + REGROTH® Dental Kit + Cytrans® granules	1-, 2-, 3-wall intra-bony defects and crater-like defects, possible furcation involvement	One group only	9	10	CAL, PPD, BOP, TM, KT, GMP, GI, PI, BDF	Clinical and radiographic improvements with respect to baseline.
[165]	**1**: Fisiograft®<br>**2**: Fisiograft® + Vicryl Mesh® membrane	2- and 3-wall intra-bony defects	Parallel	6	40	PPD, CAL, BDD, BDF	Both groups show clinical and radiographic improvements with respect to baseline, but no statistically significant differences between the groups.

[194]	**1**: OFD + PrefGel® + Emdogain®<br>**2**: OFD + PrefGel®	2- and 3-wall intra-bony defects	Split-mouth	60	15	PPD, CAL, GMP, BDD, BDF	Clinical and radiographic improvements more significant in group 1.
[195]	**1**: Simplified papillae preservation flap + PrefGel® + Emdogain®<br>**2**: Simplified papillae preservation flap	1- and 2-wall intra-bony defects	Split-mouth	6	13	CAL, PPD, GMP	Clinical improvements more significant in group 1.
[196]	**1**: Minimally invasive surgical technique + Emdogain®<br>**2**: Minimally invasive surgical technique	Intra-bony defects	Parallel	6	30	PPD, GMP, CAL, FMPS, BDD, OPG concentration, OC concentration, TGF-β1 concentration	Both groups show significant clinical and radiographic improvements with respect to baseline, but no statistically significant differences between the groups.
[197]	**1**: Minimally invasive non-surgical technique<br>**2**: Minimally invasive non-surgical technique + Emdogain®	Intra-bony defects	Parallel	12	36	BoP, PI, PPD, GMP, CAL, DR, BDD, BDAn	Both groups show significant clinical and radiographic improvements with respect to baseline, but no statistically significant differences among the groups.
[201]	**1**: 3% Hydroxypropylcellulose<br>**2**: REGROTH® Dental Kit (0.03% FGF-2)<br>**3**: REGROTH® Dental Kit (0.1% FGF-2)<br>**4**: REGROTH® Dental Kit (0.3% FGF-2)	2- and 3-wall intra-bony defects	Parallel	9	74	CAL, PPD, BOP, TM, GMP, KT, BDD	All groups show significant clinical and radiographic improvements, but no statistically significant differences among them. However, group 4 shows a significantly higher rate of increase in alveolar bone height than group 1.
[205]	**1**: 3% Hydroxypropylcellulose<br>**2**: REGROTH® Dental Kit (0.2% FGF-2)<br>**3**: REGROTH® Dental Kit (0.3% FGF-2)<br>**4**: REGROTH® Dental Kit (0.4% FGF-2)	2- and 3-wall intra-bony defects	Parallel	9	240	BDF, CAL	Significantly higher bone fill in groups 2, 3 and 4 than group 1, with group 3 giving the best results. No statistically significant differences in CAL among the groups.

[206]	**STUDY A**<br>**1**: Placebo<br>**2**: REGROTH® Dental Kit	Intra-bony defects (prevalently 2-, 2- to 3- and 3-wall)	Parallel 1:2	9	328	BDF, CAL	Significantly higher bone fill in group 2, but no statistically significant differences in CAL between the groups.
[206]	**STUDY B**<br>**1**: REGROTH® Dental Kit<br>**2**: Emdogain®<br>**3**: Flap surgery	Intra-bony defects (prevalently 2-, 2- to 3- and 3-wall)	Parallel 5:5:2	9	274	BDD, BDF, CAL	Significantly better radiographic outcomes in group 1, followed by group 2. No statistically significant differences in CAL between the groups.
[222]	**1**: PrefGel® + Emdogain®<br>**2**: HyaDENT BG®	Intra-bony defects	Parallel	24	32	CAL, PPD, GMP, BOP	Both groups show significant clinical outcomes with respect to baseline. Group 1 shows significantly higher probing depth reduction.
[227]	**1**: OFD + Gengigel® Prof syringes<br>**2**: OFD + placebo	2- and 3-wall intra-bony defects	Split-mouth	12	20	PI, GI, PPD, CAL, GMP, BDF, ACR, DR	Clinical and radiographic outcomes more significant in group 1.
[232]	**1**: Modified Widman flap + Gengigel® Prof syringes<br>**2**: Modified Widman flap + placebo	Intra-bony defects	Split-mouth	6	14	CAL, PPD, GMP, PI, BOP	Group 1 shows more significant improvements in CAL and GMP, but no statistically significant differences between the groups in the other parameters.
[233]	**1**: β-TCP<br>**2**: β-TCP + Gengigel® Prof syringes	2- and 3-wall intra-bony defects	Parallel (defect-based)	6	16	PPD, CAL, BDD	Both groups show significant clinical and radiographic improvements with respect to baseline, but no statistically significant differences between the groups.
[234]	**1**: Coronally positioned flap + Gengigel® Prof syringes<br>**2**: Coronally positioned flap	Class II furcation defects	Split-mouth	6	10	PI, GI, PPD, CAL, GMP, HBDD, BDD	Both groups show significant clinical and radiographic improvements with respect to baseline, but no statistically significant differences between the groups.

[235]	**1**: Papilla preservation flap<br>**2**: Papilla preservation flap + Ossigel®	1-, 2- and 3-wall intra-bony defects	Split-mouth	12	30	PPD, GMP, CAL, BDD	Clinical and radiographic outcomes more significant in group 2.
[238]	**1**: Ultrasonic debridement<br>**2**: Ultrasonic debridement + Aminogam®	Sites with PPD and CAL ≥ 5mm	Split-mouth	1.5	11	PPD, CAL, PI, BOP	Group 2 shows significantly improved PPD and BOP, but no statistically significant differences in the other parameters between the groups.
[241]	**1**: Laser surgery + Aminogam®<br>**2**: Laser surgery	Surgical wounds	Parallel 3:2	0.25	49	Pain, WH	Significantly improved WH in group 1. No statistically significant differnces in pain score between the groups.
[242]	**1**: Tooth extraction + Aminogam® + Condress® membrane<br>**2**: Tooth extraction + Condress® membrane	Soft tissue closure after tooth extraction	Parallel	2	40	WH, Pain, Edema, Painkillers intake	Significantly better results in group 1.
[243]	**1**: Ostim®<br>**2**: PrefGel® + Emdogain®	Intra-bony defects	Parallel	12	38	BDD, CAL, PPD, GMP, WH, FMPS, adverse events	Both groups show significant clinical and radiographic improvements with respect to baseline, but no statistically significant differences between the groups. However, patient comfort was better in group 2.
[244]	**1**: Flapless approach + PrefGel® + Emdogain®<br>**2**: Minimally invasive surgical technique + PrefGel® + Emdogain®	Intra-bony defects	Parallel	24	30	FMPS, FMBS, PPD, CAL, GMP, BDAn, BDD, Pain	Both groups have significant clinical and radiographic improvements with respect to baseline, but group 2 shows a significantly greater defect fill. However, the surgical procedure in group 2 is twice as long that in group 1.
[245]	**1**: Modified papilla preservation technique + PrefGel® + Emdogain® + DFDBA +	Intra-bony defects	Parallel	12	40	CAL, PPD, GMP, BOP, adverse events	Both groups have significant clinical improvements, but no statistically significant differences between the

	amoxicillin<br>**2**: Modified papilla preservation technique + PrefGel® + Emdogain® + DFDBA						groups. However, patient discomfort was reduced in group 1.
[246]	**1**: Coronally advanced flap + PrefGel® + Emdogain®<br>**2**: Coronally advanced flap + connective tissue graft + PrefGel® + Emdogain®	Miller Class I and Class II single gingival recession	Split-mouth	12	12	RD, RW, PRC, KT, GT, PPD, CAL	Both groups have significant clinical improvements with respect to baseline.
[247]	**1**: Coronally advanced flap + PrefGel® + Emdogain®<br>**2**: Coronally advanced flap + connective tissue graft	Miller Class I and Class II single gingival recession	Parallel	12	42	RD, KT, PPD, PRC	Both groups have significant clinical improvements with respect to baseline, but no statistically significant differences between the groups.
[248]	**1**: Granulation tissue preservation technique + PrefGel® + Emdogain®<br>**2**: Double-flap apporach with resection of the defect-filling granulation tissue + PrefGel® + Emdogain®	Intra-bony defects	Parallel	12	40	PPD, GMP, CAL, BDD	Both groups have significant clinical and radiographic improvements with respect to baseline, but group 1 shows significantly higher CAL gain in cases of noncontaining intrabony defects.
[251]	**1**: AmnioGuard membrane + NovaBone® Putty<br>**2**: BioMesh membrane + NovaBone® Putty	Intra-bony defects	Split-mouth	6	10	PPD, CAL, BDF	Clinical and radiographic results more significant in group 2.
[253]	**1**: OFD + Amnion chorion membrane<br>**2**: OFD + C-Blast™ Putty	Intra-bony defects (2- and 3-wall prevalence)	Parallel	6	22	PI, GI, PPD, CAL, BDA	Both groups show significant clinical and radiographic improvements with respect to baseline, but no statistically significant differences between the groups.

[255]	**1**: DBX® Paste<br>**2**: DBX® Putty<br>**3**: DFDBA	1-, 2-, 3-wall intra-bony defects and circumferential-type defects	Parallel	6	60	PPD, CAL, GMP, BDF, DR	All groups show significant clinical and radiographic improvements with respect to baseline, but no statistically significant differences among the groups.
[262]	**1**: Ostim®<br>**2**: Natural healing	Post extraction sockets	Split-mouth	0.5	14	Pain, Epithelialization, Biological parameters (IL-1β, Il-6, IL-10, VEGF, TGFβ2, PPARβ, BMP-4, BMP-7, ALP, OC)	Clinical improvements and biomolecular outcomes more significant in group 1.
[265]	**1**: Papilla preservation flap<br>**2**: Papilla preservation flap + Ostim®	Intra-bony defects (2-wall prevalence)	Split-mouth	6	14	BDD, PPD	Clinical and radiographic improvements more significant in group 2.
[266]	**1**: OFD<br>**2**: OFD + Ostim®	1-, 2- and 3-wall intrabony defects	Parallel	6	28	PI, GI, PPD, CAL, GMP	Clinical improvements more significant in group 2.
[290]	**1**: SRP + Placebo gel<br>**2**: SRP + 1.2% Rosuvastatin gel<br>**3**: SRP + 1.2% Atorvastatin gel	Intra-bony defects	Parallel	9	90	PI, mSBI, PPD, CAL, BDD	Clinical and radiographic improvements more significant in group 2, followed by group 3.
[291]	**1**: SRP + Placebo gel<br>**2**: SRP + 1.2% Rosuvastatin gel<br>**3**: SRP + 1% Metformin gel	Intra-bony defects	Parallel	12	90	PI, mSBI, PPD, CAL, BDD	Clinical and radiographic improvements more significant in group 2, followed by group 3.
[292]	**1**: 1.2% Simvastatin gel + Occlusive Parasorb Resodont® Forte membrane<br>**2**: 1.2% Simvastatin gel + perforated Parasorb Resodont® Forte membrane<br>**3**: PrefGel® + 1.2% Simvastatin gel + Occlusive Parasorb	2- and 3-wall intra-bony defects	Parallel	9	40	PI, GI, PPD, CAL, BDD, BDe	Clinical and radiographic improvements more significant in groups 3 and 4 than groups 1 and 2. Group 4 shows a significantly higher defect fill than the other groups.

	Resodont® Forte membrane<br>**4**: PrefGel® + 1.2% Simvastatin gel + Perforated Parasorb Resodont® Forte membrane						
[293]	**1**: Minimally invasive papilla reflection + root planing + PrefGel® + 1.2% Simvastatin gel<br>**2**: Minimally invasive papilla reflection + root planing + PrefGel® + methylcellulose gel	Defects with 6-9 mm interproximal periodontal probing depth	Parallel	12	50	PI, BOP, GMP, PPD, CAL, BDD	Both groups show significant clinical and radiographic improvements with respect to baseline. Group 1 shows significantly better CAL, PPD and BOP.
[294]	**1**: 1% Alendronate gel<br>**2**: Placebo gel	Intra-bony defects	Split-mouth	6	32	CAL, PPD, BOP, BDD	Clinical and radiographic improvements more significant in group 1.
[295]	**1**: SRP + Placebo gel<br>**2**: SRP + 1% Alendronate gel<br>**3**: SRP + Aloe vera gel	Mandibular class II furcation defects	Parallel	12	90	PI, mSBI, PPD, CAL, HCAL, BDD, DR	Clinical and radiographic improvements more significant in group 2.
[296]	**1**: SRP + Placebo gel<br>**2**: SRP + 1% Metformin gel	Intra-bony defects	Parallel	9	70	PI, mSBI, PPD, CAL, BDD, DR	Clinical and radiographic improvements more significant in group 2.

Abbreviations: ACR (Alveolar Crest Resorption), ALP (Alkaline Phosphatase), A-PRF (Advanced Platelet-Rich Fibrin), BDA (Bone Defect Area), BDAn (Bone Defect Angle), BDD (Bone Defect Depth), BDe (Bone Density), BDF (Bone Defect Fill), BDV (Bone Defect Volume), BDW (Bone Defect Width), BOP (Bleeding On Probing), CAL (Clinical Attachment Level), CGF (Concentrated Growth Factor), DR (Defect Resolution), FMBS (Full Mouth Bleeding Score), FMPS (Full Mouth Plaque Score), GI (Gingival Index), GMP (Gingival Margin Position), GSBI (Gingival Sulcus Bleeding Index), GT (Gingival Thickness), HBDD (Horizontal Bone Defect Depth), HBDF (Horizontal Bone Defect Fill), HCAL (Horizontal Clinical Attachment Level), HPD (Horizontal Probing Pocket Depth), I-PRF (Injectable Platelet Rich Fibrin), ISQ (Implant Stability Quotient), KT (Keratinized Tissue), L-PRF (Leukocyte Platelet-Rich Fibrin), MBR (Marginal Bone Resorption around implant), mSBI (Modified

Sulcus Bleeding Index), OC (Osteocalcin), OFD (Open Flap Debridement), OPG (Osteoprotegerin), PBI (Papillary Bleeding Index), PI (Plaque Index), PPD (Probing Pocket Depth), PPRAβ (Peroxisome Proliferator-Activated Receptor), PRC (Percentage Root Coverage), PRF (Platelet Rich Fibrin), PRGF (Platelets Rich in Growth Factors), PRP (Platelet Rich Plasma), RD (Recession Depth), RW (Recession Width), SRP (Scaling and Root Planing), STD (Soft Tissue Dimension), SUPRA-AG (Supra-Alveolar Attachment Gain), TM (Tooth Mobility), TP (Location of the Tip of the Papilla), T-PRF (Titanium Platelet Rich Fibrin), WH (Wound Healing Index).
